# Highly Aligned Ternary Nanofiber Matrices Loaded with MXene Expedite Regeneration of Volumetric Muscle Loss

**DOI:** 10.1007/s40820-023-01293-1

**Published:** 2024-01-04

**Authors:** Moon Sung Kang, Yeuni Yu, Rowoon Park, Hye Jin Heo, Seok Hyun Lee, Suck Won Hong, Yun Hak Kim, Dong-Wook Han

**Affiliations:** 1 Department of Cogno-Mechatronics Engineering, College of Nanoscience and Nanotechnology, Pusan National University, Busan, 46241 Republic of Korea; 2 Medical Research Institute, School of Medicine, Pusan National University, Yangsan, 50612 Republic of Korea; 3 Department of Anatomy, School of Medicine, Pusan National University, Yangsan, 50612 Republic of Korea; 4 Engineering Research Center for Color‑Modulated Extra‑Sensory Perception Technology, Pusan National University, Busan, 46241 Republic of Korea; 5 Department of Biomedical Informatics, School of Medicine, Pusan National University, Yangsan, 50612 Republic of Korea; 6 Periodontal Disease Signaling Network Research Center and Dental and Life Science Institute, School of Dentistry, Pusan National University, Yangsan, 50612 Republic of Korea; 7 BIO-IT Fusion Technology Research Institute, Pusan National University, Busan, 46241 Republic of Korea; 8 Present Address: Osstem Implant Inc., Seoul, 07789 Republic of Korea

**Keywords:** Ti_3_C_2_T_x_ MXene nanoparticle, Ternary nanofibrous matrices, Myogenesis, Regeneration of volumetric muscle loss, Next generation sequencing

## Abstract

Current therapeutic approaches for volumetric muscle loss (VML) face challenges due to limited graft availability and insufficient bioactivities. To overcome these limitations, tissue-engineered scaffolds have emerged as a promising alternative. In this study, we developed aligned ternary nanofibrous matrices comprised of poly(lactide-*co-ε*-caprolactone) integrated with collagen and Ti_3_C_2_T_x_ MXene nanoparticles (NPs) (PCM matrices), and explored their myogenic potential for skeletal muscle tissue regeneration. The PCM matrices demonstrated favorable physicochemical properties, including structural uniformity, alignment, microporosity, and hydrophilicity. In vitro assays revealed that the PCM matrices promoted cellular behaviors and myogenic differentiation of C2C12 myoblasts. Moreover, in vivo experiments demonstrated enhanced muscle remodeling and recovery in mice treated with PCM matrices following VML injury. Mechanistic insights from next-generation sequencing revealed that MXene NPs facilitated protein and ion availability within PCM matrices, leading to elevated intracellular Ca^2+^ levels in myoblasts through the activation of inducible nitric oxide synthase (iNOS) and serum/glucocorticoid regulated kinase 1 (SGK1), ultimately promoting myogenic differentiation via the mTOR-AKT pathway. Additionally, upregulated iNOS and increased NO^–^ contributed to myoblast proliferation and fiber fusion, thereby facilitating overall myoblast maturation. These findings underscore the potential of MXene NPs loaded within highly aligned matrices as therapeutic agents to promote skeletal muscle tissue recovery.

**Supplementary Information:**

The online version contains supplementary material available at 10.1007/s40820-023-01293-1.

## Highlights

- The aligned ternary nanofibrous matrices composed of poly(lactide-co-ε-caprolactone), collagen, and Ti_3_C_2_T_x_ MXene nanoparticles were fabricated (referred as PCM matrices).
- The aligned PCM matrices exhibited favorable physicochemical properties and excellent cytocompatibility and myogenic properties, which in turn promoted fast regeneration of volumetric muscle loss in vivo.
- The Ca^2+^ binding of MXene nanoparticles activated inducible nitric oxide synthase and serum/glucocorticoid regulated kinase 1-mediated mTOR-AKT pathway to promote myoblast differentiation and maturation.

**Supplementary Information:**

The online version contains supplementary material available at 10.1007/s40820-023-01293-1.

## Graphical abstract

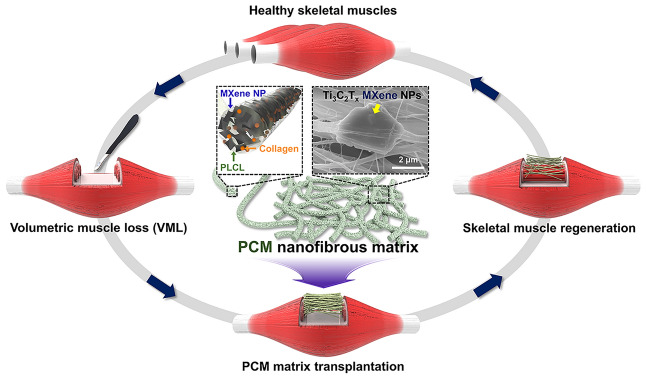

## Introduction

The regenerative capacity of the human body is frequently restricted when it comes to repairing extremely injured tissues. The decline in skeletal muscle regeneration ability with age is a primary factor, and in cases of severe injuries or diseases, extensive repairs may be necessary, surpassing the body’s innate regenerative capabilities. Especially, the military medical community has suffered from limitations for volumetric muscle loss (VML) treatments [1]. Functional free muscle transfer (FFMT) is a complex surgical technique that transplants autologous muscles to restore motor function and joint movement of injured tissues. First examined in a canine model in 1970, FFMT was subsequently utilized in clinical settings across various hospitals and has witnessed notable progress [2]. Particularly, Japan experienced significant advancements in 2011, with the introduction of pioneering approaches like single and double functional free muscle transfers, aimed at restoring upper extremity functions [3]. However, its widespread use is limited due to donor site morbidity, technical expertise requirements, and mixed results in combat-related extremity injuries [4]. Meanwhile, nonsurgical solutions such as advanced bracing strategies are utilized for treating lower extremity VML, but not for upper extremity injuries [5, 6]. Therefore, alternative approaches are required to develop new options. As one of the highlighted strategies, tissue engineering has emerged as a promising strategy by introducing bioactive acellular grafts to achieve rapid and spontaneous muscle regeneration [7]. Their main emphasis is on replicating the inherent biochemical processes between cells and niches by incorporating bioactive substances and manipulating nanotopography [8]. Recent tissue engineering strategies suggested potential applications for addressing skeletal muscle regeneration, with various skeletal muscle tissue engineering (STE) scaffolds serving as key tools to control and guide tissue regeneration [9–11]. Especially, micro or nanoscale topographical surfaces eminently highlighted in the newly emerging biochemical materials [12–14]. They are closely related to the creation of focal adhesion sites on living cells, which, in turn, prompt cytoskeleton rearrangement through mechanically activated intracellular signaling transduction (i.e., mechanotransduction) [15]. The STE scaffolds not only biochemically modulate cellular phenotype by providing biofunctional cues such as nanomaterials and growth factors, but also induce mechanotransduction by utilizing a variety of extracellular matrix (ECM) components [16].

In particular, the emergence of two-dimensional (2D) materials has gained significant attention for their potential applications as STE scaffolds because they offer the possibility of overcoming the inherent limitations of traditional muscle grafts [17]. MXene nanoparticles (NPs), a new group of 2D materials primarily composed of transition-metal carbides and nitrides, were discovered by Gogotsi et al. (2011) and have gained significant attention in the biomedical field owing to their exceptional physicochemical properties [18–21]. For instance, the impressive characteristics of MXene NPs in converting photothermal energy make them suitable for photonic hyperthermia treatment in the second near-infrared biowindow, enabling deep tissue penetration [22, 23]. Additionally, MXene NPs possess exceptional electrical conductivity and demonstrate efficient photothermal conversion, which endows them with potential applications in biosensors and bioinspired soft robotics [24, 25]. Moreover, various metal options available in MXene NPs allow them to serve as powerful contrast agents in computed tomography (CT) and magnetic resonance imaging (MRI) [26].

The motivation behind this study stems from recent reports highlighting the myogenic capabilities of MXene NPs. Li et al. demonstrated that the MXene NPs could promote reconstruction and angiogenesis of skeletal muscles by cleavage of reactive oxygen species (ROS) and alteration of proinflammatory M1 macrophages to anti-inflammatory M2 macrophages by suppressing TNF-α and IL-Iβ genes [27]. The study revealed that MXene NPs upregulated multiple myogenic genes and vascularization markers, while also demonstrating anti-inflammatory and antioxidant effects. In light of this, Boularaoui et al. conducted a study where they incorporated MXene NPs into a gelatin bioink to fabricate STE scaffolds [28]. The laden MXene NPs provided the laden C2C12 myoblasts cytocompatible environment with notable myogenic properties. We suggest that the exceptional bioactivities of MXene NPs lie in their highly customizable surface chemistry and stacked structures, which differentiate them from conventional 2D nanomaterials. The superior electrical conductivity is derived from the partially filled d-orbitals of the transition metal atoms exposed on the edges of the MXene layers during exfoliation, which support intercellular communication and cell–matrix interactions [29, 30]. Furthermore, a wide range of functional groups (–O, –OH, and –F) and ion intercalation sites within the stacked sheets could promote biomolecules avaialability of STE scaffolds [31, 32].

Despite the significant progress, the mechanisms underlying the effects of MXene NPs on myogenesis remain unclear. Currently, biological assessments predominantly concentrate on a few or dozens of markers, which hampers the comprehensive understanding of the intricate interactions between materials and cells. To address this issue, next-generation sequencing (NGS) was introduced, which involves the parallel sequencing of millions of DNA fragments, generating massive amounts of sequence data that are then analyzed to provide comprehensive insights into genetic information and biological processes [33]. Compared with conventional sequencing, NGS provides several advantages, such as high sensitivity, a wide dynamic range, quantitative gene expression information, and the ability to analyze large datasets enabling the identification of key genes, dose–response relationships, and complex patterns [34]. Therefore, we suggest that the use of NGS presents a promising opportunity to uncover the underlying mechanisms of the action of MXene NPs in myogenesis.

**Fig. 1:**
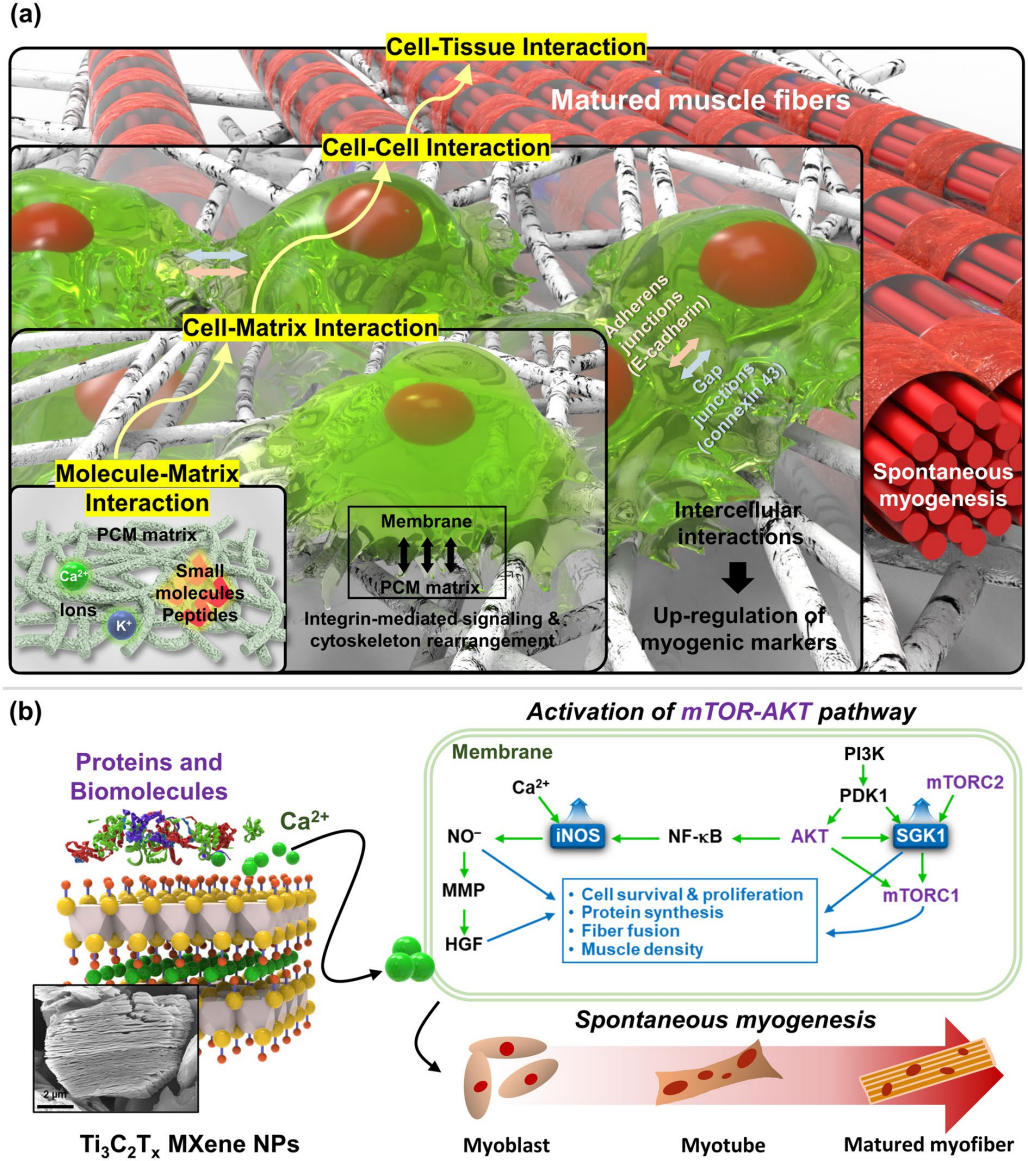
Schematic diagram of the study. **a** Bottom-up sequential interactions including molecular-matrix, cell–matrix, cell–cell, and cell-tissue interactions for myogenic differentiation of C2C12 myoblasts on PCM nanofiber matrices. **b** Adsorption of proteins, biomolecules, and ions on MXene NPs to induce mTOR-AKT signal pathway for spontaneous myogenesis of myoblasts

In this study, we fabricated biocompatible and biodegradable poly(lactide-*co-ε*-caprolactone) (PLCL) nanofibrous matrices reinforced with collagen (Col) and MXene NPs, called PCM matrices. Electrospun nanofibrous matrices were designed to provide a tailored nanotopography (e.g., aligned topography) that can efficiently mimic the morphology of the native muscular ECM. To serve as controls, three distinct compositions were prepared: pristine PLCL (P), PLCL with Col (PC), and PLCL with MXene (PM), along with each randomly oriented counterpart. The inclusion of Col, a major component of the ECM, was aimed at addressing the low cell adhesion of the PLCL matrices. A comprehensive investigation was conducted to examine the effects of the PCM nanofibrous matrices on the growth and myogenesis of C2C12 myoblasts, as evidenced by a series of immunocytochemical and genetic analyses. Furthermore, the regenerative potential was evaluated by immunohistochemical analysis after transplanting the PCM matrices into mouse VML models. NGS was used to collect and analyze the regulation of genes related to muscle regeneration. By identifying the most influential genes, the underlying mechanisms of MXene NP-induced myogenesis in C2C12 cells were proposed (Fig. 1).

## Experimental Section

### Preparation of the Exfoliated Ti_3_C_2_T_x_ MXene

2.0 g of Ti_3_AlC_2_ powder (11 Technology Co., Ltd., Jilin, China) was gradually added to 20 mL of 50% concentrated hydrofluoric acid (HF; Fisher Scientific, Fair Lawn, NJ) and magnetically stirred for 48 h at 50 °C in an oil bath. Subsequently, multilayer Ti_3_C_2_T_x_ was produced by washing with deionized (DI) water via centrifugation (3500 rpm for 3 min) and decantation several times until the pH of the supernatant reached approximately 6. 2.0 g of dry multilayer Ti_3_C_2_T_x_ was redispersed in 50 mL of deionized (DI) water, and then 40 mL of dimethyl sulfoxide (DMSO) solution was added by stirring for 24 h at room temperature. Subsequently, the aqueous suspension of Ti_3_C_2_T_x_ was filtered with DI water through nylon membrane filters and dried at room temperature to yield a delaminated Ti_3_C_2_T_x_ powder.

### Fabrication of Aligned PCM Nanofibrous Matrices

Electrospinning was employed to fabricate nanofibrous PCM matrices. A solution of PLCL (75:25, molecular weight 40–80 kDa, BMG Inc., Kyoto, Japan) and Col (Darim Tissen, Seoul, Korea) in 1,1,1,3,3,3-hexafluoroisopropanol (HFIP, Sigma-Aldrich Co., St. Louis, MO) was prepared. The concentrations of PLCL and Col in the HFIP solution were 5% and 0.5% (w/v), respectively. The concentration of PLCL and Col was optimized to avoid nanofiber aggregation or undesirable degradation in the culture environment [35, 36]. To ensure uniform distribution, the MXene solution in DI water was sonicated for 1 h. Then, the MXene solution was mixed with the PLCL and Col solution to achieve a final concentration of 400 µg mL^−1^. The mixture of PLCL, Col, and MXene (total 10 mL) was loaded into a 5-mL syringe (Henke-Sass, Wolf GmbH, Tuttlingen, Germany) equipped with a spinneret needle (inner diameter 500 µm). A high-voltage DC power supply (NanoNC, Seoul, Republic of Korea) was used at 16 kV. The working distance between the needle tip and collector was set at 9 cm, and the flow rate was maintained at 0.2 mL h^−1^. The nanofibrous matrices were collected on a steel wheel rotating at 3,000 rpm. Subsequently, the nanofibrous matrices were subjected to overnight drying under vacuum at 25 ºC to ensure complete removal of the residual solvent. Subsequently, the fabricated nanofibrous matrices were shaped into discs with a diameter of 9 mm and sterilized by overnight ultraviolet (UV) light irradiation prior to use.

### mRNA Sequencing and Identification of Differentially Expressed Genes (DEGs)

After the C2C12 myoblasts were harvested at 10 days-in-vitro, mRNA was extracted using RNeasy mini kits (Qiagen, Hilden, Germany) according to the manufacturer’s instructions. The quality of the extracted mRNA was evaluated using a Nono Chip 2100 Bioanalyzer (Agilent, Santa Clara, CA, USA). RNA-seq experiments and data analyses were conducted by Macrogen (Seoul, Republic of Korea). Purified mRNA was fragmented, and paired-end RNA sequencing was conducted using a HiSeq2000 (Illumina, San Diego, CA) sequencing system. TruSeq Stranded mRNA Sample Prep Kits (Illumina) were used to establish libraries according to the sample preparation guide**.** The quality profile of each paired-end read was checked using fastp (version 0.23.2), followed by the removal of low-quality reads and adapter contaminants. High-quality reads were mapped to the GRCm38 mouse reference genome using hisat2 (v2.2.1). The mapped gene counts were normalized using the trimmed mean of the M-values (TMM) normalization method. To identify DEGs between different nanofibrous matrices, we used the DESeq2 package of R software. We considered genes with an adjusted *p* value of less than 0.05 to have a significant differential expression between the groups. The false discovery rate (FDR) method was used to calculate the adjusted *p* value. A |log2 fold change|> 0 was considered the threshold for the cutoff value. To screen for the intersectional genes significantly expressed in each cohort, we used the R package “venn” (v1.10) to plot Venn diagrams. The Search Tool for the Retrieval of Interacting Genes (STRING) database was used to retrieve interacting genes/proteins [37]. The constructed PPI network was then displayed using Cytoscape software (version 3.8.2), from which the genes with the highest number of associations were identified as hub genes [38]. To identify the potential signaling pathways associated with MXene, we performed gene ontology (GO) enrichment analysis using the R package "clusterProfiler" (v 3.14.3) [39]. The groups with a padj of less than 0.05 and gene counts of more than two were examined.

## Results and Discussion

### Synthesis and Characterization of MXene NPs

**Fig. 2:**
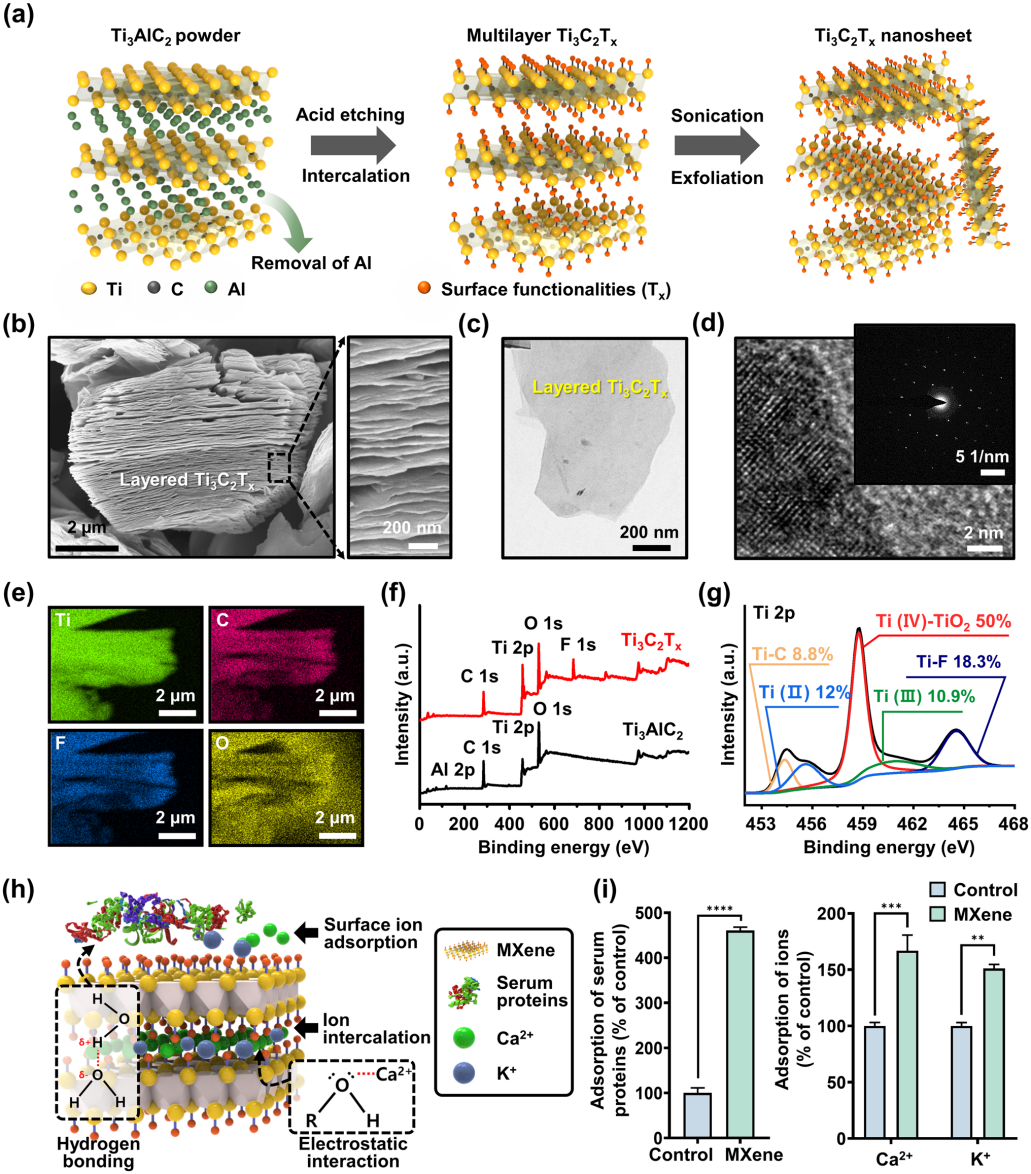
Synthesis and characterization of MXene NPs. **a** Schematic of the sequential process for synthesizing Ti_3_C_2_T_x_ MXene NP. **b** Side-view SEM image of multilayered Ti_3_C_2_T_x_ MXene after HF etching. **c** TEM image of delaminated Ti_3_C_2_T_x_ MXene NP. **d** HRTEM images; the inset image is the corresponding SAED pattern. **e** EDS mapping profile of Ti_3_C_2_T_x_, consisting of Ti (green), C (red), F (blue), and O (yellow) elements. **f** XPS spectra for Ti_3_Al_2_C_2_ and Ti_3_C_2_T_x_ powder. **g** High-resolution XPS spectrum for Ti 2p in TiC_2_T_x_ MXene NPs. **h** Schematic showing the adsorption mechanism between Ti_3_C_2_T_x_ MXene NPs and bioactive molecules such as proteins and ions. **i** Evaluation of adsorption capacity of Ti_3_C_2_T_x_ MXene for serum proteins and Ca^2+^/K^+^ ions. The data are expressed as the mean ± SD (n = 6). Asterisks (* ~ ****) denote significant differences compared to the control (*p < 0.05, **p < 0.01, ***p < 0.001, ****p < 0.0001)

Figure 2a depicts a typical process flow for preparing MXene NPs, involving sequential chemical etching and exfoliation of the layered MAX phase of Ti_3_AlC_2_. As previously reported [35], the initial step involved selective removal of the Al layer by etching the Ti_3_AlC_2_ precursor with a hydrofluoric acid (HF) solution (Fig. 2a, left). To increase the interlayer distance, DMSO was introduced into the multilayered MXene NPs (i.e., Ti_3_C_2_T_x_), where T_x_ refers to the terminal functional groups (Fig. 2a, middle). At this stage, the O atoms can readily coordinate with the Ti atoms in the Ti_3_C_2_O_2_ structure, whereas the methyl group of DMSO interacts with the O atoms of Ti_3_C_2_O_2_, facilitating electrostatic interactions that stabilize Ti_3_C_2_O_2_ during exfoliation. However, the presence of a hydroxyl group in Ti_3_C_2_(OH)_2_ impedes the interaction between DMSO and the Ti atoms, resulting in repulsive interactions between the H atom of Ti_3_C_2_(OH)_2_ and the methyl group of DMSO. Consequently, Ti_3_C_2_(OH)_2_ exfoliated with DMSO exhibited a larger interlayer distance than Ti_3_C_2_O_2_. This suggests that Ti_3_C_2_(OH)_2_ actively engages in electrostatic interactions with DMSO, promoting layer dispersion and serving as an intercalation medium for sheet formation [40]. Subsequently, ultrasonication in DI water (Fig. 2a, right) was used to produce exfoliated single- or few-layered MXene NPs, leading to surface functionalization with O, OH, and/or F groups. Through this active intercalation and separation process, a purification environment was established that transformed the MAX phase (i.e., Ti_3_AlC_2_) into Ti_3_C_2_T_x_ by removing Al and simultaneously terminating the surface with functional groups. Hence, this combinatorial reaction with simple chemistry enhances the hydrophilicity and dispersion stability in an aqueous DI water, ultimately yielding a colloidal suspension of MXene NPs.

Figure 2b shows a scanning electron microscopy (SEM) image of Ti_3_C_2_T_x_ after HF treatment, revealing the formation of separated gaps and a layered structure following the removal of the Al layer from the MAX phase. This observation demonstrates morphological similarities to the previously reported features of exfoliated graphite [41] or Ti_3_AlC_2_ [21], indicating distinct layers compared to the unreacted powders. The right image in Fig. 2b shows an enlarged view of the Ti_3_C_2_T_x_ layers, which show an average thickness of less than 12 ± 4 nm, implying a substantial specific surface area owing to the development of layer exfoliation. As presented in Fig. 2c and S1, transmission electron microscopy (TEM) images of few-layered MXene NPs display electron beam transparency, indicating their ultrathin characteristics with atomically folded, wrinkled, and overlapped features. As shown in Fig. 2d, additional high-resolution TEM (HRTEM) measurements and related selected-area electron diffraction (SAED) patterns demonstrate a highly regular arrangement of atoms with a crystalline structure in the stacked few-layered NPs comprising Ti_3_C_2_ and TiO_2_ [21]. To assess the chemical composition of the exfoliated MXene NPs, elemental scanning results indicated that the Al layer was substituted with other elements, such as Ti, C, O, and F, within the MXene structure (Fig. 2e). For detailed insight into the surface chemistry of MXene, X-ray photoelectron spectroscopy (XPS) analysis was performed, as shown in Fig. 2f, g. A comparison of the XPS spectra of the as-prepared Ti_3_C_2_T_x_ with those of the source MAX phase shows that the Ti_3_C_2_T_x_ spectrum clearly exhibits the presence of Ti, F, O, and C, whereas the Al atoms are selectively removed and no longer appear in the spectrum (Fig. 2f). The high-resolution XPS spectra in Fig. 2g and S2 confirm successful elemental substitution, with a specific focus on the Ti 2*p*, C 1*s*, and termination groups (i.e., F 1*s* and O 1*s*). In the Ti 2*p* spectrum, the binding energies (BE) of Ti–C, Ti (II), and Ti (IV)–TiO_2_ were 454.3, 455.6, and 458.7 eV, respectively. Moreover, the dominance of the Ti–O bond was observed in the case of the O end group for HF-exfoliated Ti_3_C_2_T_x_ [40]. As shown in Fig. S2, the high-resolution XPS spectrum of the C 1*s* for the MXene powder exhibited four major peaks corresponding to Ti–C, C–C, C–O, and C–F at 281.1, 284.8, 285.2, and 288.6 eV, respectively. Notably, the presence of C–O bonds can be attributed to MXene oxidation, which resulted in the formation of TiO_2_ and a network of carbon atoms. After the exfoliation process, the C 1*s* spectrum of MXene indicated that the surface was primarily functionalized with –OH groups, accompanied by graphitizing carbon and carbides. Based on the collected dataset, we found that the produced MXene NPs possess multifunctional surface properties with elemental configurations that encompass functional groups such as –F, –OH, and –O_x_.

Regarding the synthetic production of MXene NPs, a significant limitation arises from the necessity of using strong acids for the etching process, which is essential for breaking the metallic bond between M and A [21, 42, 43]. Alternative sequential processes have been developed to effectively generate individually delaminated MXene NPs; however, these processes can introduce atomic defects that serve as crucial sites for oxidation reactions [44]. Nevertheless, the partial oxidation reactions lead to the presence of various functional groups, including Ti–O and Ti–Ox, in the Ti_3_C_2_T_x_ structure. These functional groups offer advantages in terms of serving as cell scaffolds and facilitating interactions with proteins, ions, or biomolecules, as depicted in Fig. 2h. The quantity of proteins or the concentration of ions in the extracellular microenvironment can influence cellular responses such as adhesion, proliferation, and metabolism [45, 46]. In other words, MXene NPs present numerous opportunities to enhance cell function owing to their abundance of functional groups, making them promising bioactive materials. In particular, they can serve as suitable substrates capable of inducing interactions through hydrogen bonding and electrostatic forces with serum proteins such as bovine serum albumin (BSA) [47]. Furthermore, recent studies have revealed that the numerous hydroxyl groups (e.g., Ti–O or Ti–OH groups) in MXene NPs act as adsorption sites for ions such as Ca^2+^, Mg^2+^, Na^+^, and K^+^, resulted by electrostatic attraction-induced electrical adsorption [48–50]. In light of this, we investigated the adsorption capacity of MXene NPs for serum proteins and ions, specifically Ca^2+^ and K^+^ (Fig. 2i). Notably, after incubation for 24 h, a significant increase in the adsorption rate was detected for the MXene NPs, that is, ~ 460% for serum protein, 166% for Ca^2+^, and 155% for K^+^, in comparison to the control group (i.e., untreated MXene NPs). These findings strongly suggest that MXene NP has the beneficial ability to interact with physiologically active substances, which can profoundly influence the behavior of adjacent cells, including adhesion, migration, proliferation, and differentiation, thereby confirming its potential as a cell culture biomaterial.

### Fabrication and Characterization of PCM Nanofibrous Matrices

**Fig. 3:**
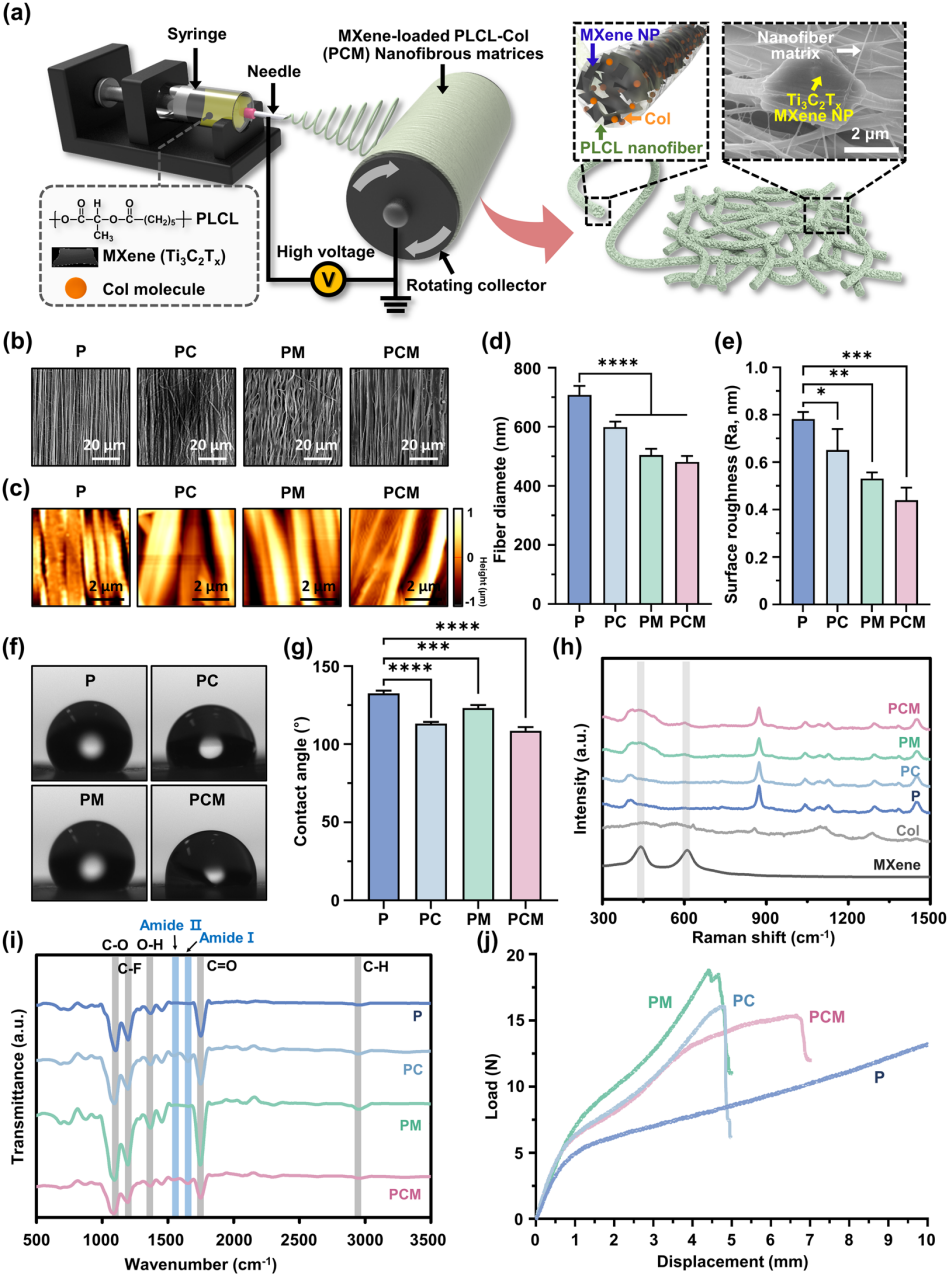
Fabrication and characterization of PCM nanofibrous matrices. **a** Schematic diagram of electrospinning process. **b** FE-SEM and **c** AFM images indicating the surface morphology of nanofibrous matrices. Quantification of **d** fiber diameters and **e** surface roughness based on FE-SEM and AFM images, respectively. **f** Macroscopic images of sessile drops on each matrix and corresponding **g** contact angles. **h** Raman spectra and **i** FT-IR spectra denoting specific peaks of P, PC, PM, and PCM nanofibrous matrices. **j** Load–displacement curves of P, PC, PM, and PCM nanofibrous matrices. The data are expressed as the mean ± SD (n = 6). Asterisks (* ~ ****) denote significant differences compared to the control (*p < 0.05, **p < 0.01, ***p < 0.001, ****p < 0.0001). Scale bars denote 20 µm for (**b)** and 2 µm for (**a)** and (**c)**

To reinforce PLCL matrices with Col and MXene NPs, nanofibrous matrices were prepared using a typical electrospinning process (Fig. 3a). The mass ratio of MXene NPs in the nanofibrous matrices was carefully optimized based on the cytotoxicity assessment. The cytocompatibility of MXene NPs is a significant factor to consider when creating a scaffold system because the excessive uptake of NPs can hinder the normal growth of cells. Therefore, the impact of MXene NPs on the metabolic activity and membrane integrity of C2C12 myoblasts was thoroughly examined at the beginning of cell culture. This was done using standard assays such as the Cell Counting Kit-8 (CCK-8) assay (Fig. S3a) and lactate dehydrogenase (LDH) release assay (Fig. S3b). The CCK-8 assay revealed that cell viability decreased as the concentration of MXene NPs increased up to 250 µg mL^‒1^ after 24 h and 48 h of incubation (Fig. S3a). Notably, a correlation was observed between a significant reduction in cell viability (≤ 80%) and specific concentration ranges of 125–250 µg mL^‒1^ after 24 h and 62.5–250 µg mL^‒1^ after 48 h. In addition, a markedly high level of intracellular LDH release (≤ 150%) was observed at 250 µg mL^‒1^ after 24 h and 125–250 µg mL^‒1^ after 48 h indicating that excessive doses of MXene NPs impede the membrane integrity of C2C12 cells (Fig. S3b) [51]. The efficiency of myogenesis was evaluated by immunocytochemical analysis after 3 and 7 days of incubation with different concentrations of MXene NPs (Fig. S4a, b). With an increase in the concentration of MXene NPs, the expression of myosin heavy chain (MHC), a well-known marker for early myogenesis [52, 53], increased up to MXene NPs of 20 µg mL^‒1^. To ensure the prevention of the toxicity of MXene NPs and achieve the maximum myogenic effect, 20 µg mL^‒1^ was chosen as the final optimal concentration. Considering these findings and earlier study [35], along with the volume of the polymer solution and size of the nanofibrous sheet (which had a disc shape with a diameter of 9 mm), the mass ratio of the MXene NPs in the specified area was inferred to be within the optimized range.

The physicochemical properties of the nanofibrous matrices were characterized as follows: In our experimental scheme, compositional nanofibrous matrices were set with P, PC, PM, and PCM in two different orientations (aligned vs. random) to differentiate the roles of additives and the topographical cues. The randomly oriented nanofibrous matrices exhibited an irregular structure, whereas the aligned nanofibrous matrices exhibited a perpendicular orientation (Fig. S5a, b). When the surface morphology was examined using scanning electron microscopy (SEM), highly aligned networked nanofibers were observed (Fig. 3b). These morphological features were designed to resemble the longitudinally aligned perimysial collagen cables in skeletal muscle matrices with interconnected microporous structures [54]. We measured the size changes; the average fiber diameters for each sample of P, PC, PM, and PCM were 708 ± 30, 599 ± 18, 504 ± 21, and 481 ± 20, respectively (Fig. 3d). When MXene NPs, Col, or both were added into the PLCL matrices, the fiber diameter decreased by 84–67% compared to that of the PLCL matrices. This result can be attributed to the delicate intramolecular interactions derived from the incorporation of MXene NPs and Col, which alter the flow dynamics of the electrospinning solution, which in turn determines the diameter and morphology of the electrospun nanofibers. In our case, we concluded that the addition of Col and MXene NPs lowers the viscosity of the PLCL solution because the hydrophilic components tend to have a stronger attraction to water molecules, which can lead to an increase in the fluidity of the liquid [55]. Previous studies have shown that nanofibrous matrices of specific sizes can significantly impact cell behavior and myogenic differentiation. For example, nanofibers in the 305–667 nm range enhanced cell adhesion, mobility, and proliferation [56], while 436 nm-sized nanofibrous matrices supported myogenic differentiation of human mesenchymal stem cells (hMSCs) and increased key myogenic markers [57]. In this study, adding Col and MXene NPs to the electrospinning solution reduced fiber size from 708 ± 30 to 481 ± 20 nm, suggesting potential benefits for myogenesis due to size-dependent topographical effects.

The surface topography and roughness of the nanofibrous matrices were explored using atomic force microscopy (AFM) (Fig. 3c). The measured surface roughness (Ra) of P, PC, PM, and PCM nanofibrous matrices were 0.782 ± 0.03, 0.652 ± 0.089, 0.531 ± 0.026, and 0.44 ± 0.054 μm, respectively (Fig. 3e). The reduction in fiber diameter with the addition of Col and MXene NPs is considered to be the dominant factor for the surface roughness decrease. The close packing of nanofibers with smaller diameters induced a smoother surface, as previously reported [36]. Subsequently, the hydrophilicity of each substrate was evaluated by measuring the water contact angle using the sessile droplet method (Fig. 3f, g). Compared with P, the PC, PM, and PCM exhibited significantly decreased contact angle (i.e., increased hydrophilicity), suggesting that the addition of Col and MXene NPs can enhance the bioactivity of scaffolds [58, 59]. Furthermore, the physicochemical properties of the aligned nanofibrous matrices were similar to those of the randomly oriented matrices, indicating that the alignment process during fabrication did not significantly impair their inherent properties (Fig. S6a–d).

Raman and Fourier-transform infrared (FT-IR) spectroscopies were used to determine the chemical composition of the nanofibers. In Fig. 3h, two major Raman peaks at 440 and 610 cm^−1^ (labeled with gray columns) were observed for the MXene NPs, which can be assigned to ω_6_ and ω_3_, respectively [60]. Because different chemical groups are attached to the surface of Ti_3_C_2_T_x_, a comprehensive interpretation of the experimental Raman spectra must take into account the influence of hybridized vibration modes of –F and –OH [61]. To further investigate the chemical characteristics of the matrices (Fig. 3i), the bands of C–O (1090 cm^−1^), O–H (1384 cm^−1^), C-H (2990 cm^−1^), and C = O (1757 cm^−1^) corresponding to PLCL were observed (labeled with gray columns) [62, 63]. In addition, amide I and amide II bands from Col were observed near 1657 and 1552 cm^−1^ (labeled with a blue column). MXene-specific peaks at 1084, 1178, and 1452 cm^‒1^ derived from C–O, C–F, and O–H, respectively, were observed in the MXene-incorporated groups [64, 65].

The flexibility of a nanofibrous matrix depends on its failure strain, which means that a higher strain corresponds to greater flexibility, resulting in a stronger fibrous matrix [66]. By incorporating Col and MXene NPs, the yield displacement and yield load of the matrices were improved, indicating an increase in their stiffness (Fig. 3j and S7a–c) [67]. The addition of Col and MXene NPs to nanofibrous matrices can induce mechanical reinforcement and reduce the fiber diameter, which results in increased flexibility and overall strength [66]. Therefore, incorporating Col and MXene NPs can be considered an appropriate approach for producing durable and resilient grafts suitable for skeletal muscle tissues that experience repetitive contraction and relaxation. The Young’s modulus of prepared nanofibrous matrices was 84–224 kPA, which was significantly increased by the incorporation of MXene NPs and Col (Fig. S7d). Furthermore, the range is similar to or slightly higher than that of native skeletal muscle ECM (approximately 8–80 kPa) suggesting the PCM matrix can provide proper mechanical stimulation to seeded cells to induce their intrinsic phenotypes [68].

To evaluate the biodegradability, we conducted an in vitro degradation test (Fig. S8). When immersed in DPBS at 37 °C for 21 days, all groups displayed degradation rates ranging from 4.8 to 7.8% by weight, with no significant differences between the groups (Fig. S8a). However, the accelerated degradation test allowed to distinguish the degradation characteristics of the nanofibrous matrices (Fig. S8b) [69]. After 7 days, PC (56.82%, weight loss compared to the initial state) and PCM (48.34%) exhibited higher degradation rates in comparison to P (30.43%) and PM (27.45%). This difference can primarily be attributed to the hydrophilic properties of uncrosslinked collagen. Furthermore, the rapid degradation rate observed in the PCM matrices under accelerated conditions suggests their feasibility for decomposition in an in vivo environment.

### Cell Behaviors of C2C12 Myoblasts on PCM Nanofibrous Matrices

**Fig. 4:**
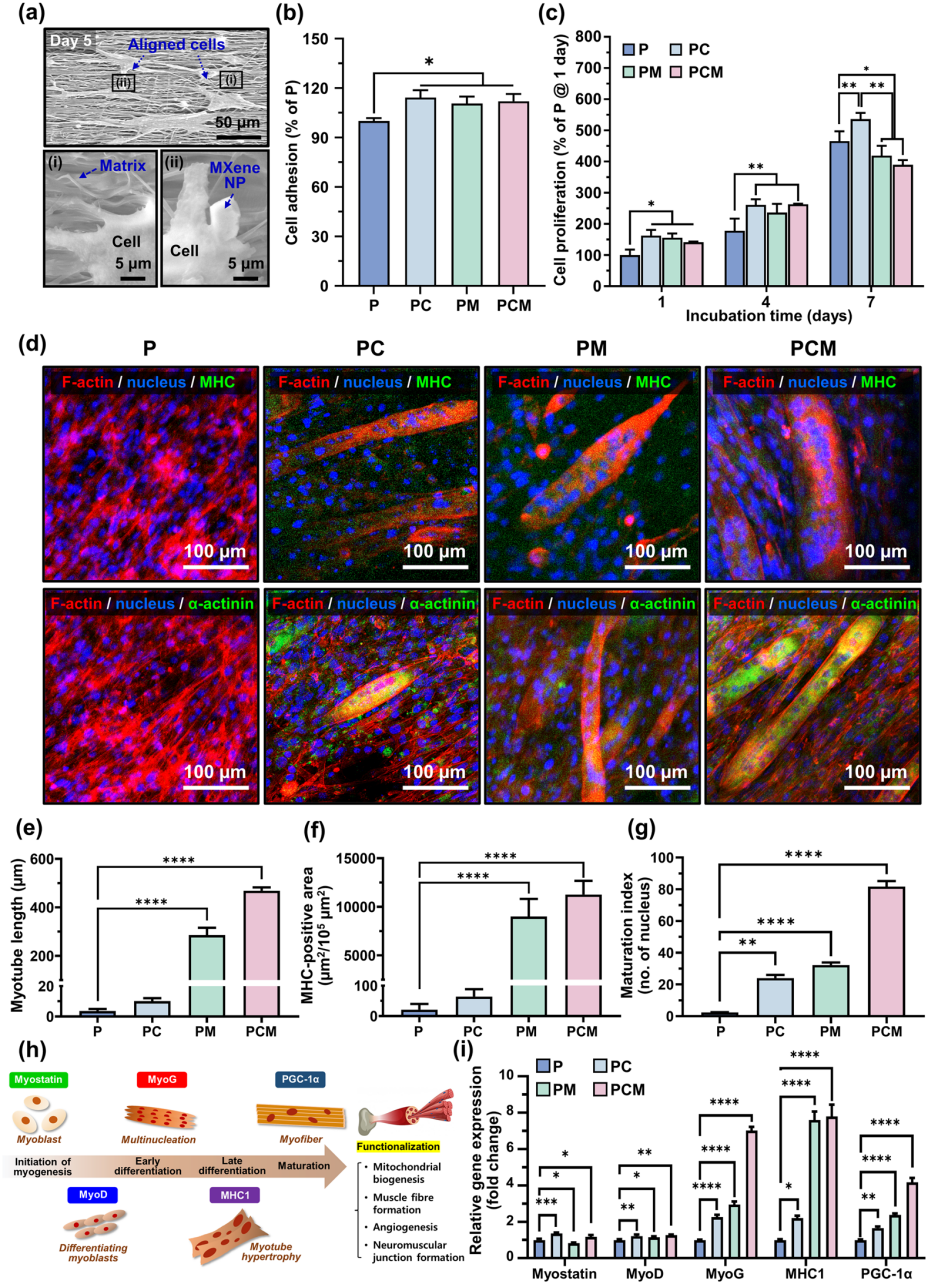
Cell behaviors of C2C12 myoblasts on PCM nanofibrous matrices. **a** SEM images of C2C12 myoblasts on PCM nanofibrous matrices after 5 days of culture. **b** Cell adhesion after 24 h of seeding and **c** cell proliferation during 7 days of culture. **d** Immunocytochemical analysis on C2C12 myoblasts after 14 days of incubation on P, PC, PM, and PCM nanofibrous matrices. F-actin and nucleus were stained with tetramethylrhodamine (TRITC)-labeled phalloidin (red) and 4’,6-diamidino-2-phenylindole (DAPI) (blue), whereas MHC (upper row) and α-actinin (lower row) were stained with fluorescein isothiocyanate (FITC) (green). Quantified data for **e** myotube length, **f** MHC-positive area, and **g** maturation index. **h** Schematic diagram and **i** qRT-PCR results of myogenic marker regulation in P, PC, PM, and PCM nanofibrous matrices. All fluorescence images were obtained from representative samples. Asterisks (*–****) denote significant differences compared to the control (*p < 0.05, **p < 0.01, ***p < 0.001, ****p < 0.0001). Scale bars are as follows: 5 or 50 µm for (**a**) and 100 µm for (**d**)

The morphology of the C2C12 myoblasts on the PCM nanofibrous matrices was characterized using SEM (Fig. 4a). The C2C12 myoblasts spread their filopodia and came into direct contact with the nanofibers and MXene NPs. In addition, the cell attachment and proliferation of C2C12 myoblasts on the P, PC, PM, and PCM nanofibrous matrices were assessed using a CCK-8 assay. The initial adhesion of C2C12 myoblasts was significantly increased (*p* < 0.05) in the PC, PM, and PCM groups, and no significant differences among the PC, PM, and PCM groups were observed (Fig. 4b).

The population of myoblasts consistently increased during the culture period in all the nanofibrous matrices (Fig. 4c). After 7 days of incubation, the PC matrices showed significantly increased (*p* < 0.01) proliferation compared to the P, PC, and PM groups. In addition, the enhanced adhesion and proliferation in Col-incorporated groups were similarily observed on the randomly oriented nanofibrous matrices (Fig. S9). The proliferation of PM and PCM possibly decreases owing to the initiation of early myogenesis, which induces multinucleation while withdrawing the proliferation cycle. On the other hand, the inclusion of Col in the matrix enhances its hydrophilicity, thereby regulating the expression of the αvβ3 integrin subunit. Moreover, type I Col itself serves as a specific binding site for α1β1 and α2β1 integrins [70]. Although MXene NPs facilitate cell–matrix interactions by supporting focal adhesion formation with filopodia protrusion and activating integrin-mediated signaling pathways, the hydrophilic surface nature of Col was presumably the dominant factor rather than that of MXene NPs.

In addition, immunocytochemical analysis was conducted on C2C12 myoblasts cultured on the P, PC, PM, and PCM nanofibrous matrices (Fig. 4d). In the PC, PM, and PCM, the long-aligned multinucleated cells exhibited representative myogenic markers, myosin heavy chain (MHC), and sarcomeric α-actinin [71]. Compared to their random counterpart, aligned nanofibrous matrices induced more MHC expression by topographical cues (Fig. S10). Particularly, a high degree of alignment in the PCM may maximize the contractile power of the muscle, which is conveyed by the acceleration of myogenic differentiation. The presence of α-actinin, a protein found in the Z line of the sarcomere within the skeletal muscle, provides compelling evidence for the functional development and structural stability of the Z line, which plays a crucial role in maintaining the contractile power and integrity of muscle fibers. These quantitative data further support the notion that the PCM matrices exhibit the highest level of myogenic activity in C2C12 cells. This is evident from the significant increase in myotube length, MHC-positive area, and maturation index in the cultured myotubes (Fig. 4e–g).

Cell cycle progression is often influenced by cytoskeleton remodeling, which is regulated by cell–matrix interactions and various signaling pathways, including G1-phase cyclin-dependent kinases (CDKs) and focal adhesion kinases (FAKs) [72]. In our study, we observed that MXene NPs induced the reorganization of the cytoskeleton in C2C12 cells, leading to the clustering and alignment of actin filaments. This phenomenon resulted in spontaneous myogenic differentiation of the cells, even in the absence of exogenous myogenic inducers. The effects of MXene NPs on cytoskeletal remodeling and differentiation were confirmed by western blotting (Fig. 4h, i). Notably, the expression levels of myostatin and myogenic differentiation 1 (MyoD) were upregulated in the PCM group compared with those in the control group, indicating that the initiation of myogenesis was influenced by Col and MXene. Furthermore, the early and late differentiation markers myogenin (MyoG) and MHC1 showed significantly higher expression levels (7.02- and 7.8-fold, respectively) in the PCM group than in the control group, suggesting that MXene NPs had a remarkable impact on multinucleation and myotube hypertrophy. Peroxisome proliferator-activated receptor-γ coactivator-1α (PGC-1α), a marker associated with myofiber morphogenesis and muscle function, including mitochondrial biogenesis, muscle fiber formation, angiogenesis, and neuromuscular junction formation, exhibited substantial upregulation (4.17-fold) in the PCM group. Furthermore, these trends significantly reinforced by aligned surfaces whem comparing the marker expression on the randomly oriented matrices (Fig. S11). Based on these findings, we hypothesize that PCM nanofibrous matrices support the entire myogenic differentiation process in C2C12 cells.

### Immunohistochemical Analysis on Mouse Muscle Defect Models After Transplantation of PCM Nanofibrous Matrices

**Fig. 5:**
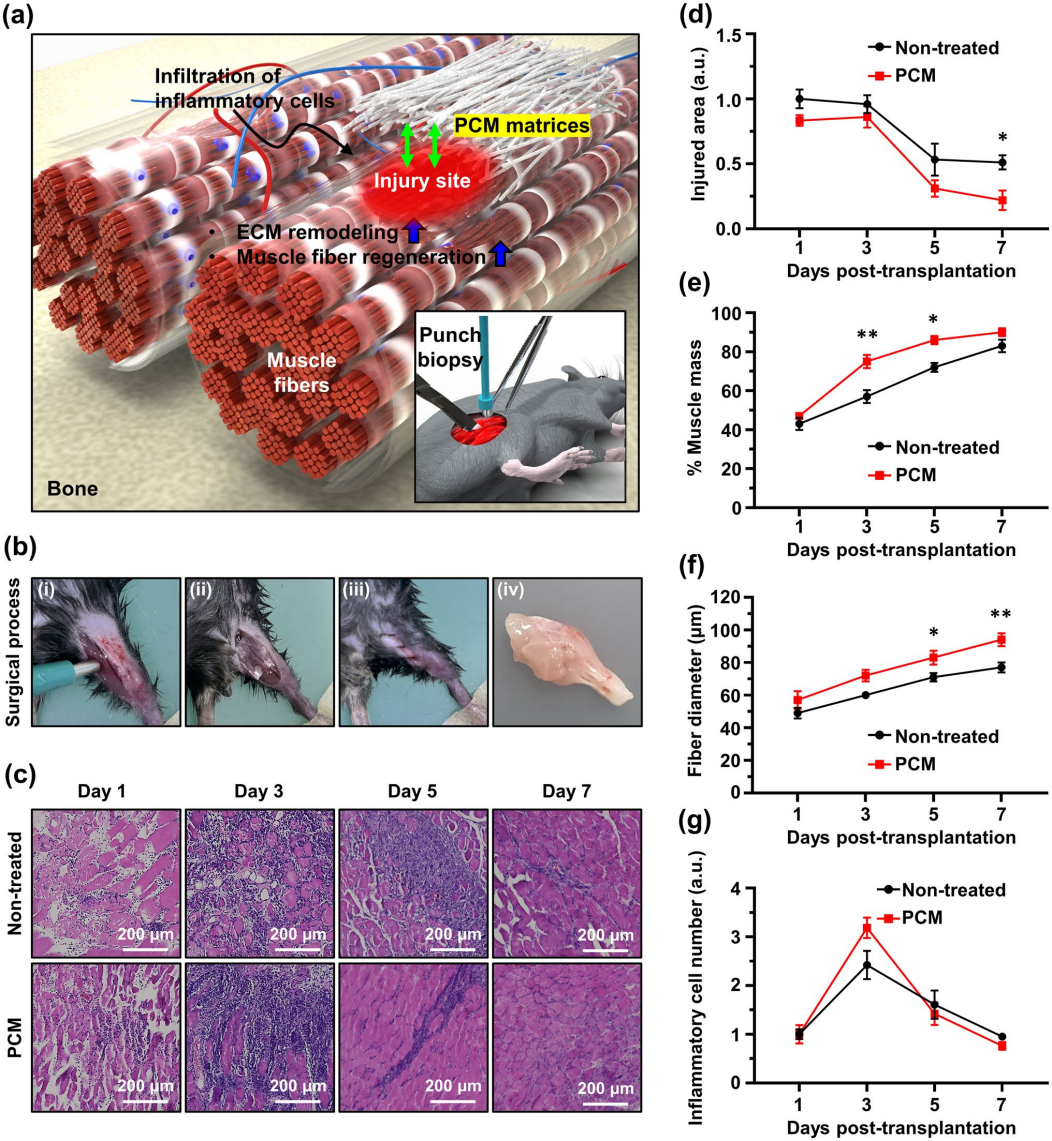
PCM mediated muscle regeneration in a mouse model of VML. **a** Schematic diagram of VML injured TA muscle of a mouse. **b** 2 mm biopsy punch induced muscle loss in mice. (i-iv) surgical process and extracted tissues. **c** The TA muscle cross sections were stained with H&E to visualize nucleus (purple) and cytosol (pink). **d** VML injury area, **e** muscle mass, **f** muscle fiber diameter, and **g** inflammatory cell number were quantified from H&E staining images. All images were obtained from representative portions of the samples. Asterisks (* ~ **) denote significant differences compared to the control (*p < 0.05, **p < 0.01). Scale bars of **c** denote 200 µm

To assess the effect of PCM on muscle recovery, a surgical procedure was performed to induce volumetric muscle loss (VML) in the rat tibialis anterior (TA) muscles. Subsequently, the extent of muscle regeneration was evaluated over a period of 7 days (Fig. 5a, b). Mouse tissue sections were subjected to hematoxylin and eosin (H&E) staining to assess the extent of muscle recovery in both PCM-treated and untreated mice at 1, 3, 5, and 7 days post-injury. After five days, the PCM-treated mice exhibited a significant increase in the total number of muscle cells compared with the non-treated group, indicating enhanced muscle remodeling and recovery (Fig. 5c). We also conducted a comparative analysis of injury recovery after one week across different treatment groups, non-treated, P, PC, PM, and PCM, in the mouse VML model. PCM exhibited the most substantial degree of injury recovery. The extent of muscle cell regeneration after injury was assessed based on measurements of the injured area, muscle mass, fiber diameter, and inflammatory cell count. Notably, the injured area decreased more rapidly in the PCM group (Fig. 5d). Moreover, the muscle mass showed a pronounced increase in the PCM group on day 3. Although a slightly higher value was observed on day 7, the difference compared to that in the non-treated group was minimal (Fig. 5e). The PCM-treated samples exhibited consistently higher values of the fiber diameter than the non-treated group (Fig. 5f). Additionally, the number of immune cells displayed an apparent increase relative to that in the non-treated group up to the third day after the PCM treatment. However, from the fifth day onward, the number of immune cells remained lower than that in the non-treated group (Fig. 5g). The collagen deposition was evaluated with each group of the VML model through Masson’s trichrome stain. Seven days after surgery, more collagen deposition (blue color) was observed in the PCM group (Fig. S12). We found that the application of PCM significantly promoted the regeneration of muscle fibers and mechanical function recovery after VML injury.

The surgical transplantation of STE scaffolds has been found to facilitate volumetric regeneration and functional muscular activation in both small and large animal models [73, 74]. Sicari et al. reported de novo formation of skeletal muscles through the utilization of STE scaffolds, which create an inductive niche for the recruitment and differentiation of endogenous myogenic progenitor cells in both the preclinical and clinical studies [75]. In addition, Mase et al. reported the case of using extrinsic scaffolds in patients who experienced extremity trauma with VML [76]. These STE scaffold-mediated responses occur through various mechanisms, including the provision of biofunctional cues to surrounding myoblasts, as well as the recruitment of stem/progenitor cells through the formation of chemotactic cryptic peptides and the modulation of macrophage phenotype [77, 78]. Consequently, the surgical insertion of STE scaffolds into the VML region has shown to be particularly effective when combined with rehabilitation methods. A study by Gentile et al. has shown that the surgical transplantation of scaffolds in the VML area, along with early mobilization and intensive rehabilitation, results in greater functional improvements compared to rehabilitation alone [79]. The observed clinical enhancements following the implementation of STE scaffolds and rehabilitation align with numerous preclinical studies that have demonstrated the ability of tissue engineering to stimulate a positive remodeling response after VML [74, 80]. Based on these findings, we propose that the introduction of PCM nanofibrous matrices could be a promising clinical approach for VML recovery.

### Mechanism for MXene-Enabled Spontaneous Myogenesis Through NGS

#### DEG Analysis and Functional Enrichment Analysis

**Fig. 6:**
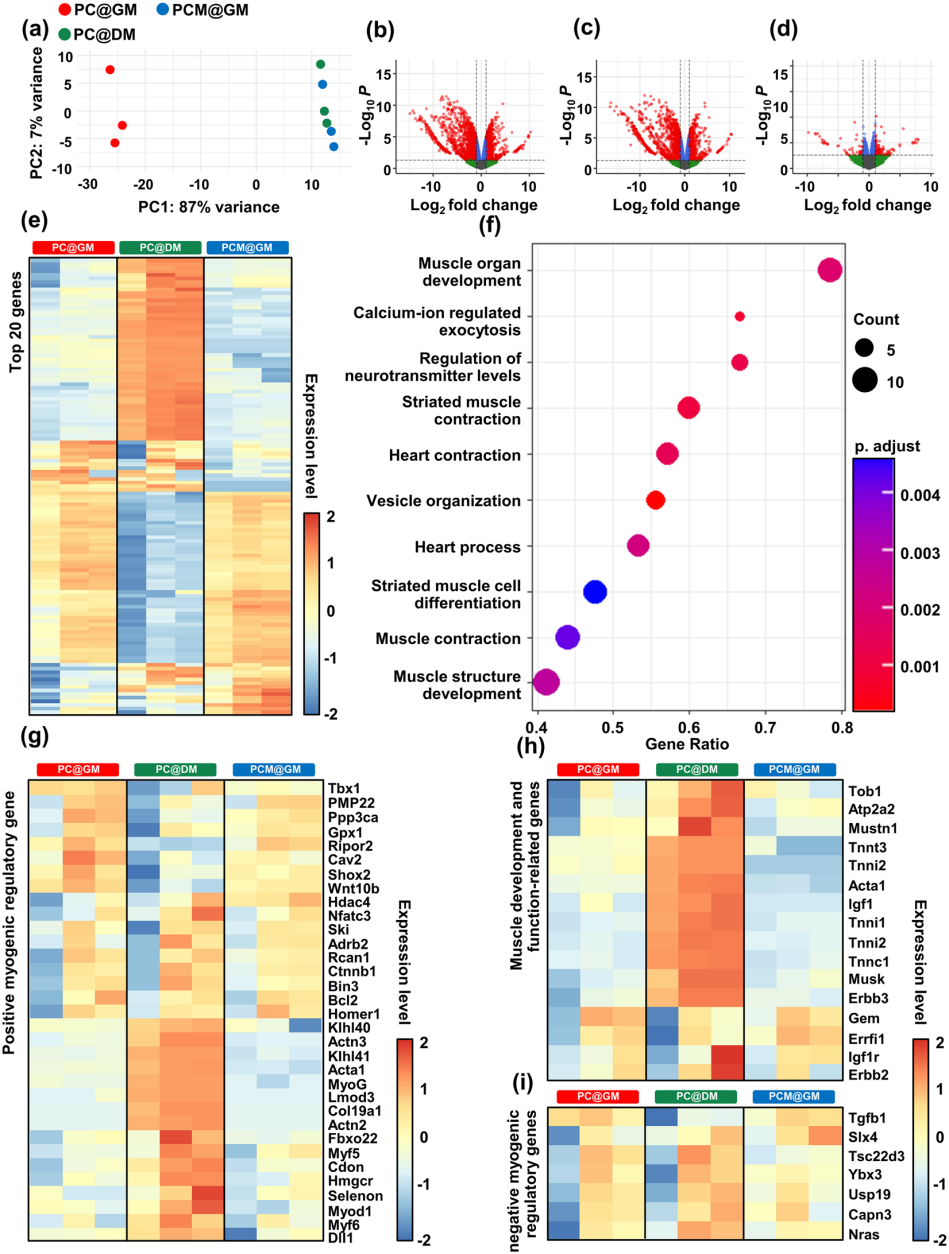
Differential transcriptome profiling of C2C12 cells cultured on PC@GM, PC@DM, and PCM@GM for 10 days. **a** A plot generated by principal component analysis illustrating the variance observed in RNA-seq data, specifically obtained through DESeq2 log-normalized RNA-seq analysis. The plot depicts the percentage of variance accounted for by each component. **b**–**d** A volcano plot exhibiting the differential expression of genes in C2C12 cells and comparing the following conditions: **b** PC@GM vs. PC@DM, **c** PC@DM vs. PC@GM, and **d** PC@GM vs. PCM@GM. **e** Heatmaps showing the top 20 DEGs in different groups. Detailed names of genes are denoted in Fig. S13. The color-coding is based on log-transformed read count values. **f** Gene ontology terms for biological processes associated with DEGs between PC@DM and PCM@GM cultured C2C12 cells. **g** A heatmap showing the expression patterns of positively regulated myogenic regulatory genes across different groups. **h** A heatmap illustrating the expression patterns of genes related to muscle development and function across different groups. **i** A heatmap highlighting the expression patterns of negatively regulated myogenic regulatory genes across different groups. Heatmap labels are as follows: Left column (red) for PC@GM, middle column (green) for PC@DM, and right column (blue) for PCM@GM

To investigate the impact of MXene NPs on muscle cell regeneration at the genetic level, C2C12 myoblasts were seeded on PC matrices and subjected to various medium conditions, including growth medium (GM) and differentiation medium (DM) as controls, which were abbreviated as PC@GM and PC@DM. Similarly, the experimental group consisted of cells seeded on the PCM and cultured with GM (abbreviated as PCM@GM). Subsequently, cellular samples were collected, and RNA sequencing was conducted to analyze the gene expression patterns and identify the genes modulated by MXene NPs. Principal components analysis (PCA) was performed to assess the overall patterns across the samples (Fig. 6a). A total of 2916 DEGs between PC@GM and PC@DM were screened according to the above criteria, including 1173 and 1143 genes upregulated in PC@GM and PC@DM, respectively (Fig. 6b). A total of 2374 DEGs between PC@DM and PCM@GM, including 823 genes upregulated in PC@DM and 1,551 in PCM@GM, were screened (Fig. 6c). In total, 166 DEGs were identified between PC@GM and PCM@GM, including 109 genes upregulated in PC@GM and 57 in PCM@GM (Fig. 6d). The number of DEGs suggests that adding MXene to the PC@GM medium cause less genetic changes between PCM@GM and PC@GM compared to what is observed in the PC@DM medium. Nevertheless, it’s important to highlight that PCM@GM has a stronger ability to promote muscle cell differentiation compared to PC@GM, suggesting that MXene can promote the expression of genes that can induce muscle cell differentiation. The top 20 genes in each analysis were visualized using a heap map (Fig. 6e). A gene ontology analysis was conducted by comparing the genes that exhibited differences between PC@GM and PCM@GM. Our findings revealed that biological processes associated with muscle development, including muscle organ development and calcium ion-regulated exocytosis, were enriched (Fig. 6f). Myogenic differentiation was assessed by examining the expression of several myogenic genes, including desmine (des), myostatin, myogenic factor 5 (myf5), MyoD, MyoG, troponin I (tnni), and MHC1 (Fig. 6g). These genes regulate cell differentiation, myogenesis, and force generation [81–83]. Among the myogenic regulatory genes, nine showed elevated expression levels in both PC@GM and PCM@GM, surpassing those observed in PC@DM. Conversely, 16 genes exhibited pronounced expression in the PC@DM group. Additionally, we analyzed genes involved in muscle development and function (Fig. 6h) as well as negative regulators of myogenic differentiation, myogenesis, and muscle regeneration (Fig. 6i). The PCM@GM medium is a composite medium composed of PC@GM supplemented with MXene NPs. Although the overall gene expression profile closely resembled that of the PC@GM, the specific gene expression patterns associated with muscle cell regeneration and differentiation were similar to those of the PC@DM. These discernible expression patterns imply that MXene NPs may exert their influence on muscle development and regeneration through mechanisms distinct from those of PC@DM.

#### Inducible Nitric Oxide Synthase (iNOS) and Serum/Glucocorticoid Regulated Kinase 1 (SGK1) Promote Muscle Regeneration Through MXene NPs

**Fig. 7:**
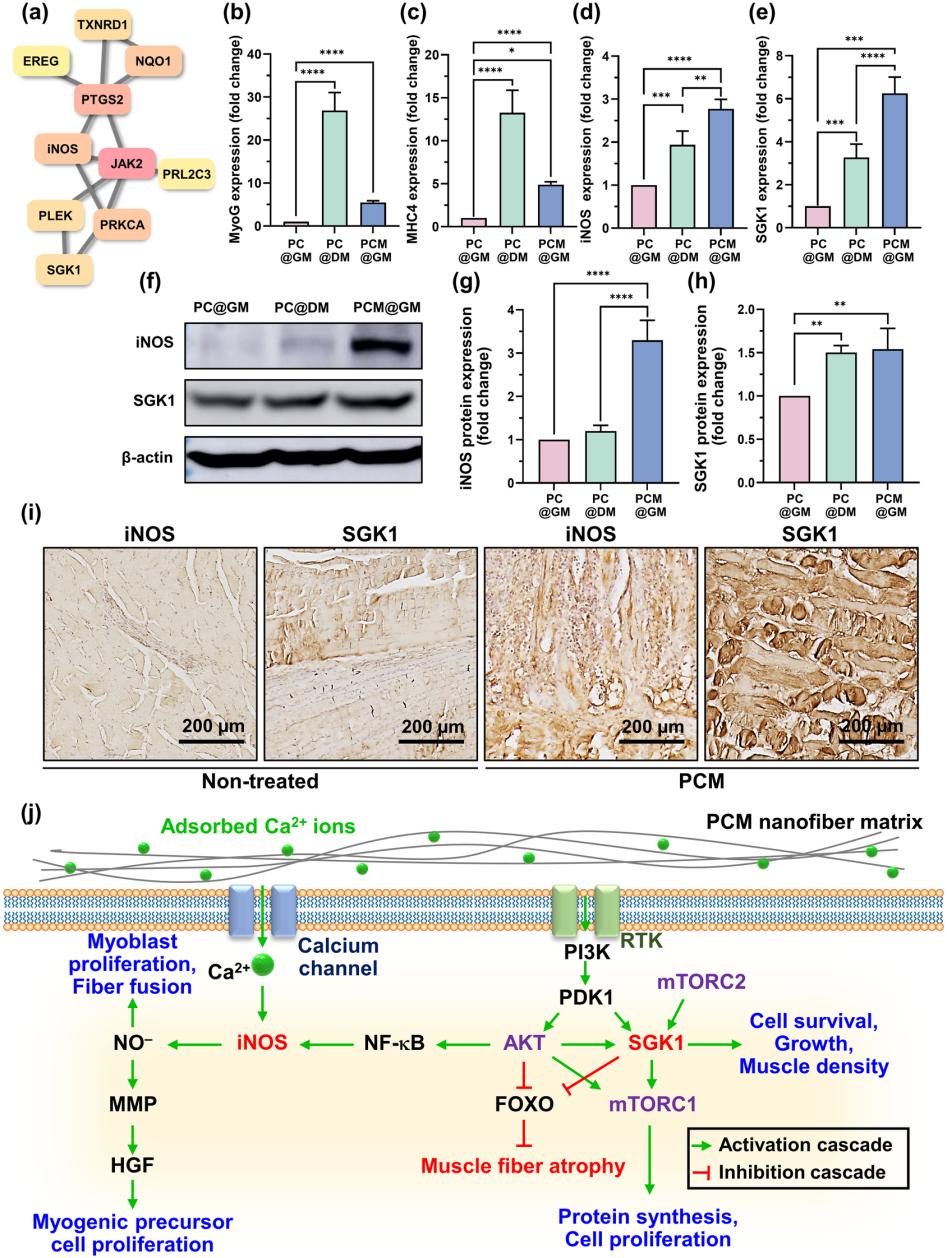
Effect and mechanism of PCM@GM-mediated muscle regeneration in an in vivo model. **a** A total of 10 hub genes with higher degrees of hubness were selected and filtered into the PPI network complex using the STRING database and Cytohubba. **b**–**e** MyoG, MHC4, iNOS and SGK1 expression levels during C2C12 muscle differentiation were determined using qRT-PCR in the PC@GM, PC@DM, and PCM@GM groups. **f**–**h** Protein levels of iNOS and SGK1 were evaluated by western blotting. **i** VML-injured TA muscles were sectioned and stained with iNOS and SGK1 antibodies. **j** Schematic representation of Ca^2+^ induced iNOS and SGK1 and muscle regeneration. All images were obtained from representative portions of the samples. Asterisks (* ~ ****) denote significant differences compared to the control (*p < 0.05, **p < 0.01, ***p < 0.001, ****p < 0.0001). Scale bars of **i** denote 200 µm

A total of 42 genes exhibiting higher expression levels in PCM@GM relative to those in PC@DM and PC@GM were selected. These genes were subjected to string and hub gene analyses to identify 10 genes associated with high humidity (Fig. 7a). Subsequently, experimental investigations were conducted to elucidate the relationship between MXene NPs and the expression of inducible iNOS and serum/glucocorticoid-regulated kinase 1 (SGK1), which are closely associated with calcium signaling and muscle regeneration [84–86]. RNA expression analysis confirmed that MyoG and MHC4, which are involved in muscle regeneration [87], exhibited the highest levels in the PC@DM group. However, iNOS and SGK1 showed the highest expression levels in the PCM@GM group (Fig. 7b–e). Changes in protein expression were validated using western blot analysis, which confirmed a significant increase in iNOS and SGK1 levels in the PCM@GM group. These findings are consistent with the observed upregulation of iNOS and SGK1 expression in PCM@GM, as detected by qRT-PCR (Fig. 7f–h). Immunohistochemistry revealed that SGK1 and iNOS expression in the muscles of PCM@GM mice increased compared with that in non-treated mice (Fig. 7i).

Calcium is an important ion that plays a key role in muscle regeneration [88], and its regulation is associated with the activity of several signaling pathways, including the iNOS and SGK1 signaling pathways. When calcium forms a complex with calmodulin, this calcium-calmodulin complex serves as an activator for a range of enzymes, including specific isoforms of NOS. SGK1 can undergo calcium-mediated activation through various mechanisms, including calcium/calmodulin-dependent kinase kinase (CaMKK) and calcium/calmodulin-dependent kinase IV. Elevations in intracellular calcium levels trigger the activation of CaMKK, subsequently resulting in the phosphorylation and activation of SGK1 [89]. SGK1 is a protein kinase involved in the regulation of various cellular processes, including muscle regeneration. SGK1 plays a role in regulating the activity of downstream targets involved in muscle growth and repair, such as skeletal muscle mass and the formation of new muscle fibers [85, 90–92]. iNOS plays a role in orchestrating the different phases of muscle repair and regulating the immune response. iNOS can be denoted as an M1 macrophage marker. In prior investigations, MXene has demonstrated its capacity to induce the transformation of M1 macrophages into the M2 phenotype, and concurrently reduce the inflammatory response in the injured area [27]. Our experimental findings indicate that the quantity of proinflammatory cells remains unchanged, both with and without PCM treatment, throughout the post-injury recovery period. Therefore, changes in muscle-related markers led us to focus our study on the association between iNOS expression and myoblast differentiation [93]. MXenes demonstrate remarkable antioxidant capacity attributed to their structural characteristics. They possess abundant surface functional groups like –F, –OH, and –O, which enhance their reactivity with ROS. Additionally, MXenes’ electron-rich nature allows them to efficiently pair with electron-deficient free radicals [27]. Moreover, the alternating C–Ti layers in MXene, held together by weak van der Waals forces, readily react with ROS [94]. This inherent ability to capture ROS contributes to their strong antioxidant potential. As inflammation and oxidative stress are interconnected processes, MXenes’ antioxidant properties may alleviate oxidative stress, subsequently reducing inflammation. Regarding the potential immunogenicity and immune modulation effect of MXene, the paragraph highlights that MXene has antioxidant properties and can modulate excessive inflammatory responses in damaged muscle areas. This modulation of inflammation and oxidative stress may contribute to an overall anti-inflammatory effect, as observed in vivo and in vitro. Therefore, MXene appears to have the potential to influence the immune microenvironment by promoting a more favorable balance of inflammatory responses, which can be discussed as part of its immune modulation effect. However, further studies and discussions specifically addressing the immunogenicity of MXene would be needed to provide a comprehensive analysis of its impact on the immune system.

iNOS is responsible for the production of nitric oxide (NO), which plays a role in muscle regeneration. Calcium signaling modulates iNOS expression in myoblast cells [95]. Elevated intracellular calcium levels resulting from mechanical stress or injury can activate calcium-dependent signaling pathways that lead to increased iNOS expression [96]. In skeletal muscle regeneration, the intricate interplay among NO, matrix metalloproteinases (MMPs), and hepatocyte growth factor (HGF) plays a pivotal role in orchestrating satellite cell responses [97]. Satellite cells, which are quiescent stem cells residing within the skeletal muscle tissue, undergo activation, proliferation, differentiation, and fusion with existing muscle fibers upon encountering muscle damage, thereby facilitating muscle regeneration and repair. NO contributes to satellite cell activation, whereas MMPs play a critical role in ECM remodeling by fostering a conducive microenvironment for satellite cell migration and differentiation [98]. Furthermore, HGF acts as a growth factor that attracts satellite cells to the site of injury and promotes their proliferation and differentiation [99].

In this study, we proposed an explanatory model for MXene-induced cell regeneration. MXene NPs promote the deposition of calcium in surrounding cells, leading to elevated intracellular Ca^2+^ levels in myoblast cells. The subsequent increase in Ca^2+^ signaling triggers the upregulation of iNOS and SGK1. Enhanced SGK1 activity influences cell proliferation, survival, and muscle cell regeneration by stimulating the mTOR-AKT pathway. In order to investigate potential alterations in gene expression within the mTOR-AKT pathway, we evaluated the levels of *AKT*, *PI3K*, and *mTOR* genes. PI3K represents a key signaling pathway (i.e., PI3K/Akt) involved in the initiation of skeletal muscle differentiation by inducing cell differentiation and hypertrophy, and the suppression of PI3K activity is known to disrupts the differentiation process in rat and mouse skeletal muscle cell lines [100, 101]. As a downstream effector and regulator of AKT, mTOR molecule regulates mRNA translation and metabolism for maintenance and regeneration of skeletal muscles [102, 103]. Our findings affirm that expressions of AKT (1.3-fold), PI3K (1.9-fold), and mROT (2.0-fold) were signifcantly up-regulated in PCM@GM groups compared to PC@GM groups, suggesting the capability of MXene NPs in activating PI3K/AKT/mTOR signaling pathway to facilitate myogenesis (Fig. S14a–c). Furthermore, the upregulated iNOS and the resulting increase in NO^−^ contribute to myoblast proliferation and fiber fusion, ultimately promoting matured myotube formation (Fig. 7j). Finally, restoration of neuromuscular functions in P, PC, PM, and PCM-transplanted mice was estimated by grip strength test (Fig. S15a). At days 1 and 3 post-transplantation of each nanofibrous matrices, there was no significant differences between groups (Fig. S15b). However, at 7 days post-transplantation, pulling forces were significantly increased at PM and PCM groups compared to non-treated groups. In detail, 74.72% in PM and 89.19% in PCM of grip strengths were restored compared to the time prior to the surgical procedure, suggesting the motor function of VML was significantly restored by MXene NPs.

## Conclusions

In this study, we developed electrospun PCM nanofibrous matrices to evaluate their potential as STE scaffolds for VML regeneration. The results indicated that the PCM nanofibrous matrices exhibited structural similarity to the natural ECM and excellent physicochemical properties, which can provide favorable microenvironments for the unprecedented cellular behavior of C2C12 myoblasts. As designed in our scheme, the PCM nanofibrous matrices enhanced the initial cell adhesion, proliferation, and myogenic differentiation. Furthermore, in vivo assays revealed that PCM-treated mice exhibited enhanced muscle remodeling and recovery, suggesting a significant promotion of muscle fiber regeneration and mechanical function recovery with the application of PCM after VML injury. The NGS results showed distinct gene expression patterns between the different treatment groups, with genes related to muscle development and calcium-ion-regulated exocytosis enriched in the PCM nanofibrous matrices. Additionally, the expression of myogenic genes involved in cell differentiation and force generation was significantly upregulated in the PCM matrices, suggesting that MXene NPs can influence muscle regeneration through a series of processes. We propose a model that the MXene NPs promote calcium deposition around the cells, leading to elevated levels of intracellular Ca^2+^, which triggers the upregulation of iNOS and SGK1. Enhanced SGK1 activity influences cell proliferation, survival, and myogenesis by activating the mTOR-AKT pathway. Additionally, upregulated iNOS and increased production of NO^−^ contribute to myoblast proliferation and fiber fusion, ultimately promoting overall myotube maturation.

Proof-of-concept studies delving into living cell responses with matrices are essential for developing effective scaffolds that can be implemented in clinical applications. The aligned PCM nanofibrous matrices could provide biophysical cues to convert extracellular stimuli into intracellular biochemical signals via mechanosensing and mechanotransduction [104]. Sequential signal transduction generated from actin cytoskeleton alignment with nanotopography can guide oriented morphogenesis, migration, and specific myogenic behaviors [105]. Accordingly, aligned nano/micro topographical models have offered substantial benefits to provide contact guidance as a myoblast culture platforms [106, 107]. Together with regulation of mechanical strain and gradient stiffness under the controllable external fields, aligned nanofibrous matrices are expected to enhance in vivo muscle regeneration over traditional therapeutic strategies. On the other hand, our presented work has limitation in terms of using C2C12 cell lines instead of human primary myoblasts. Although using cell lines could be useful for basic research due to their homogeneity and consistency in phenotypes, future works should be validated by experiments with primary cells to avoid cell lines genetic and epigenetic vulnerability to other stimuli [108].

Recent advancements in nanobiotechnology have proposed possibilities for personalized grafts contributing paradigm shifts in regenerative medicines. Nonetheless, because MXene NPs are infancy in the biomedical field, there remain substantial limitation between laboratory testing and clinical application, necessitating additional investigation to bridge this gap. First of all, exhaustive toxicological investigation in both in vitro and in vivo models should be analytically evaluated. Although studies demonstrated MXene NPs has relatively low cytotoxicity and good biocompatibility compared to other NPs, comprehensive pharmacokinetic characteristics should be further elucidated including organ distribution, clearance pathway, interaction with cells or drugs, long-term genotoxicity, hemotoxicity, and immune responses using various in vitro and in vivo models. In addition, the field of nanomedicine holds great promise for the biomedical and pharmaceutical industry, but it currently lacks adequate regulatory guidance despite calls from the research community. The first step depends on the regulatory bodies, including National Institute of Health (NIH), the European Science Foundation and the European Technology Platform, to coordinate in order to establish consistent international definitions and clarity on NPs (especially, MXene) in human uses [109]. In addition, it’s essential to estabilish robust protocols for standardized fabrication process ensuring their sterility and storage conditions. In conclusion, we anticipate that further research and exploration of MXenes will enhance our comprehension and unveil additional possibilities for their clinical translations, ultimately contributing to the well-being of humans.

## Supplementary Information

Below is the link to the electronic supplementary material.

Supplementary file1 (PDF 2219 KB)
