# Supplementary material for: Highly Aligned Ternary Nanofiber Matrices Loaded with MXene Expedite Regeneration of Volumetric Muscle Loss
Source: Nanomicro Lett. 2024 Jan 4;16:73. doi: 10.1007/s40820-023-01293-1 (PMC10767178; doi:10.1007/s40820-023-01293-1)
Supplement: Supplementary file 1 — Supplementary file1 (PDF 2219 KB) [file 40820_2023_1293_MOESM1_ESM.pdf]

Supporting Information for

Highly Aligned Ternary Nanofiber Matrices Loaded with MXene Expedite Regeneration of Volumetric Muscle Loss

Moon Sung Kang[1, †], Yeuni Yu[2, †], Rowoon Park[1, †], Hye Jin Heo[3], Seok Hyun Lee[1, a)], Suck Won Hong[1, 4, *], Yun Hak Kim[2, 5, 6, *], Dong-Wook Han[1, 7, *]

[1] Department of Cogno-Mechatronics Engineering, College of Nanoscience and Nanotechnology, Pusan National University, Busan 46241, Republic of Korea

[2] Medical Research Institute, School of Medicine, Pusan National University, Yangsan 50612, Republic of Korea

[3] Department of Anatomy, School of Medicine, Pusan National University, Yangsan 50612, Republic of Korea

[4] Engineering Research Center for Color-Modulated Extra-Sensory Perception Technology, Pusan National University, Busan 46241, Republic of Korea

[5] Department of Biomedical Informatics, School of Medicine, Pusan National University, Yangsan 50612, Republic of Korea

[6] Periodontal Disease Signaling Network Research Center & Dental and Life Science Institute, School of Dentistry, Pusan National University, Yangsan 50612, Republic of Korea

[7] BIO-IT Fusion Technology Research Institute, Pusan National University, Busan 46241, Republic of Korea

[a)] Present address: Osstem Implant Inc., Seoul 07789, Republic of Korea

[†] Moon Sung Kang, Yeuni Yu, and Rowoon Park contributed equally to this work.

*Corresponding authors. E-mail: swhong@pusan.ac.kr (S.W.H.); yunhak10510@pusan.ac.kr (Y.H.K.); nanohan@pusan.ac.kr (D.-W.H.)

S1 Experimental Procedures

S1.1 Microscopic observations and elemental analysis on MXene nanoparticles (NPs)

The surface morphologies of the prepared MXene NPs and all the nanofibrous matrices were observed by field-emission scanning electron microscopy (FE-SEM, Supra 40VP, Zeiss, Oberkochen, Germany) at an accelerating voltage of 15 kV. The crystallinity and elemental mapping of the MXene NPs were analyzed using scanning transmission electron microscopy with energy-dispersive X-ray spectroscopy operated at 200 kV (STEM-EDS; Talos F200X, Thermo Fisher Scientific, Waltham, MA). Chemical analysis of MXene NPs was conducted by X-ray photoelectron spectroscopy (XPS) (AXIS Supra, Kratos Analytical Ltd., Manchester, UK) to confirm the C 1s, O 1s, F 1s, Al 2p, and Ti 2p states.

S1.2 Adsorption of ions and biomolecules on MXene NPs

During each experiment, cells were washed one to three times with Dulbecco's phosphate-buffered saline (DPBS). The adsorption of serum proteins was measured using fetal bovine serum (FBS) (Welgene, Daegu, South Korea). 1 mL of FBS was reacted with 1 mg of MXene NPs pellet in 15-mL tubes for 24 h in a humidified incubator at 37 °C with a 5% carbon dioxide (CO_2) atmosphere. After rinsing with DPBS, the Pierce™ bicinchoninic acid (BCA) protein assay kit (ThermoFisher Scientific)

was used to assess the quantity of adsorbed proteins on the MXene NPs. Briefly after the chelation of copper with protein in an alkaline environment to form a light blue complex, BCA reacts with the reduced cation to form an intense purple BCA/copper complex. The absorbance was measured at 562 nm using a microplate reader (Varioskan LUX; Thermo Fisher Scientific). Similarly, the quantity of ion adsorption and intercalation into MXene NPs was measured using a colorimetric calcium assay kit (Abcam, Cambridge, UK) and a fluorometric potassium assay kit (Abcam), according to the manufacturers' protocols. During the calcium assay, a chromogenic complex was created by the interaction between calcium ions and 0-cresolphthalein. The absorbance of the complex was measured using a microplate reader at a wavelength of 575 nm. In the potassium assay, the detection agent facilitates the reaction between two substrates in the presence of potassium. This reaction generated an intermediate product that subsequently reacted with the developer. As a result, the non-fluorescent probe was oxidized, leading to the production of a robust and persistent fluorescence signal. The fluorescence yield at excitation and emission wavelengths of 535 and 587 nm, respectively, was measured using a microplate reader.

S1.3 Morphological analysis of PCM nanofibrous matrices

Four types of nanofibrous matrices were prepared: poly(lactide-co-ε-caprolactone) (PLCL), collagen (Col)-incorporated PLCL, MXene-incorporated PLCL, and both MXene- and Col-incorporated PLCL, abbreviated as P, PC, PM, and PCM, respectively. FE-SEM was used to observe the surface morphology of the nanofibrous matrices at an accelerating voltage of 15 kV. The topography of the nanofibrous matrices was analyzed through atomic force microscopy (AFM, NX10, Park Systems, Suwon, South Korea) at 25 ºC in the air. AFM imaging was conducted in the non-contact mode, utilizing a multi-silicon scanning probe with a resonant frequency of approximately 300 kHz. The resulting images were analyzed using the XEI software (Park Systems). The water contact angles of the matrices were determined by the sessile drop method using a contact angle measurement system (SmartDrop; Femtofab, Seongnam, South Korea). A 1 µL sessile drop of distilled water was placed on each matrix for measurement. Fiber orientation was quantified using the FFT mode of the ImageJ software (National Institutes of Health, Bethesda, MD) based on the FE-SEM images.

S1.4 Chemical analysis of nanofibrous matrices

Fourier transform infrared (FT-IR) spectroscopy (Spectrum GX, PerkinElmer, Waltham, MA) and Raman spectroscopy (NS220, Nanoscope Systems, South Korea) were used for compositional analysis of the matrices. The FT-IR spectra were recorded in the absorption mode within the wavelength range of 500–3500 cm^{-1}, employing a resolution of 4.0 cm^{-1} and 16 scans. Raman spectra of each sample were separately characterized using a Raman spectrometer (laser wavelength = 532 nm and laser power of 5 mW) at the range of 300–1500 nm.

S1.5 Mechanical analysis of nanofibrous matrices

The PCM nanofibrous matrices were cut into 5 mm × 10 mm rectangular samples and attached to paper windows using double-sided tape. Tensile properties of matrices were determined using a universal materials tester (H5 K-S, Hounsfield, UK) at 20 °C and a humidity of 65% using a 10 N load cell at a crosshead speed of 5 mm min^{-1}. The thicknesses of the samples were measured with a digital micrometer with a precision of 1 µm. Determination of Young's modulus (E) was calculated as follows:

$$E = (K * Y)/A$$

Where K is stiffness, Y is yield displacement, and A is sample area.

The degradation rates of the nanofibrous matrices in an in vitro environment were evaluated in the following process. Rectangular samples measuring 1 cm × 5 cm were immersed into 10 mL of DPBS for pre-determined time periods. These samples were subjected to incubation either at 37°C to mimic physiological conditions or at 70°C to accelerate the process, using a shaking incubator. Following the reaction, the samples were dried at 25°C for 48 h. Their weights were then measured, and the change in weight from their initial state was calculated as the remaining weight percentage.

S1.6 In vitro cell culture conditions

C2C12 murine myoblast cells were purchased from the American Type Culture Collection (ATCC, Rockville, MD). Cells were routinely cultured in a growth medium (GM) composed of Dulbecco's Modified Eagle Medium (DMEM, Welgene) supplemented with 10% FBS and 1% antibiotic-antimycotic solution (Abs, Sigma-Aldrich, St. Louis, MO), which contained 10,000 units of penicillin, 10 mg of streptomycin, and 25 µg of amphotericin B per mL. As a positive control, after 48 h of culture, GM was replaced with differentiation medium (DM), which was composed of DMEM supplemented with 2% horse serum (HS, Welgene) and 1% Abs. Cells were cultured in a humidified incubator at 37 °C with a 5% CO_2 atmosphere. Fresh culture media were replaced every 48 h, and the cells were subcultured at 80% density. For in vitro experiments, cells were seeded at a density of 5×10^4 cells/sample in 48-well plates (unless specifically mentioned otherwise) and assessed after incubation for each predetermined period. During each experimental process, the cells were washed one to three times with DPBS.

S1.7 Cytocompatibility of MXene NPs on C2C12 myoblasts

The cytotoxicity of MXene at different doses was evaluated using the Cell Counting Kit-8 assay (CCK-8, Dojindo, Kumamoto, Japan). The C2C12 myoblasts were seeded in 96-well plates at a density of 1.5×10^4 cells/well. They were then exposed to increasing concentrations of MXene NPs (0–500 µg mL^{-1}) for 24 and 48 h. The culture medium was replaced with a ten-times diluted CCK-8 assay solution and incubated for 2 h at 37 °C in a humidified incubator. After incubation, the supernatants were collected and transferred to 96-well plates. Absorbance was measured at 450 nm using a microplate reader. To assess the integrity of cell membranes, a lactate dehydrogenase (LDH) assay kit (Sigma-Aldrich) was used to measure the amount of LDH that leaked out of the cells. C2C12 myoblasts were seeded in 96-well plates at a density of 1.5×10^4 cells/well and treated with increasing concentrations of MXene (ranging from 0 to 500 µg mL^{-1}) for 24 and 48 h. After transferring the supernatant to a new 96-well plate, the LDH assay solution was added as per the manufacturer's protocol, and the plate was incubated for 30 minutes at 25 °C in darkness. The absorbance was measured at 490 nm using a microplate reader.

S1.8 Immunocytochemical analysis on MXene-treated C2C12 myoblasts on tissue culture plastic (TCP)

The nucleus, F-actin, and myosin heavy chain (MHC) in C2C12 myoblasts were visualized by immunofluorescence staining. After pre-determined periods of culture, the cells were fixed with a 3.7% formaldehyde solution (Sigma-Aldrich) for 10 min, treated with 0.1% Triton X-100 (Sigma-Aldrich) for 5 min, and blocked with a 2% bovine serum albumin (BSA; GenDEPOT, Barker, TX) solution for 30 min. MHC was immunostained with an anti-MHC antibody (Abcam), and after an overnight reaction at 4 °C, secondary goat anti-rabbit IgG heavy and light chains (Abcam) conjugated with fluorescein isothiocyanate (FITC) were reacted for 1 h. Subsequently, the nucleus and F-actin were counterstained with 1 µM of 4,6-diamidino-2-phenylindole (DAPI) (Sigma-Aldrich) and 165 nM of tetramethylrhodamine isothiocyanate (TRITC)-labeled phalloidin (Molecular Probes, Eugene, OR). Fluorescent images were captured using a fluorescence microscope (IX 81, Olympus, Tokyo, Japan).

S1.9 Assessment of cellular behaviors of C2C12 myoblasts cultured on nanofiber matrices

FE-SEM was used to visualize the morphology of the C2C12 myoblasts cultured on the PCM matrices. Cell adhesion and proliferation were measured using a CCK-8 assay kit, as described previously. Immunocytochemical analysis of cells cultured on the nanofibrous matrices for ten days was conducted using a confocal laser scanning microscope (CLSM, LSM800, Zeiss). Additionally, the anti-MHC antibody (Abcam) or anti-sarcomeric α-actinin antibody (Abcam) was used to visualize the sarcomere structures of mature myotubes. For image analysis and processing, we used the Fiji software, an open-source image processing package based on ImageJ. The myotube length, MHC-positive area, and maturation index were quantified based on the presented immunofluorescence or low-magnification images.

S1.10 Quantitative real-time polymerase chain reaction (qRT-PCR)

After 10 days of culture, the total RNA was extracted using the RNeasy Mini Kit (Qiagen, Hilden, Germany). Complementary DNA (cDNA) was synthesized using a Smart Gene Compact cDNA

Synthesis Kit (Smart Gene, South Korea). qPCR was performed using the LightCycler 96 Real-Time PCR System (Roche, Basel, Switzerland). The primer sequences can be found in Table S1, including myostatin, myogenic differentiation 1 (MyoD), myogenin (MyoG), myosin heavy chain 1 (MHC 1), peroxisome proliferator-activated receptor-γ coactivator-1α (PGC-1α), myosin heavy chain 4 (MHC4), inducible nitric oxide synthase (iNOS), and serum/glucocorticoid-regulated kinase 1 (SGK1). Target mRNA expression relative to the housekeeping gene expression (GAPDH) was calculated using the ΔΔCT method.

S1.11 Western blotting

Cells were collected, homogenized in protein lysis buffer, and centrifuged at 13,000 rpm for 10 min at 4 °C. After centrifugation, 30 µg of protein was loaded onto a 10% sodium dodecyl sulfate-polyacrylamide gel and electrophoretically separated. Proteins were then transferred onto nitrocellulose membranes. The membranes were blocked with 5% skim milk and incubated with antibodies against iNOS and SGK1 (ABclonal Technology, Woburn, MA) overnight at 4 °C (1:500 dilution). The membranes were probed with an anti-β-actin antibody (ABclonal Technology) for 3 h (1:1000 dilution) as an internal control. Antigen-antibody complexes were detected using chemiluminescence (Thermo Fisher Scientific).

S1.12 Animal tests condition

All animal procedures were approved by the Animal Care Committee of the Pusan National University (PNU-2023-0284). Male C57BL/6 mice, aged 8 weeks, were housed in an animal house (SPF degree) with a barrier. The mice were sedated using 3% isoflurane and maintained under anesthesia with 1–1.5% isoflurane.

S1.13 Volumetric muscle loss (VML) injury model

To establish VML on the tibialis anterior (TA) muscle, a normal TA muscle was injured using a biopsy punch (2 mm in diameter). The injured TA muscle area was covered with the PCM matrices. Tissue regeneration in the wound was evaluated at the designated time points.

S1.14 Immunohistochemical analysis and grip test

To observe muscle histology, paraffin-embedded tissue sections of 10 µm thickness were collected and stained with hematoxylin and eosin (Abcam) following the manufacturer's instructions. For immunohistochemistry, tissue sections were incubated overnight with a polyclonal antibody against iNOS and SGK1 (Abcam; 1:100 dilution) at 4 °C. Immunoreactions were visualized using the EnVision Detection System Kit (Dako, Agilent, Santa Clara, CA), and nuclei were stained with Mayer's hematoxylin solution. Muscle tissue was stained with Masson's Trichrome Stain Kit (Abcam) following the manufacture's instruction. After staining, images were captured using an AxioScan Z1 digital slide scanner (Zeiss) and analyzed using Zen Blue software (Zeiss). ImageJ software was used to obtain quantitative data such as injured area, muscle mass, fiber diameter, and inflammatory cell numbers from the H&E images. The grip strength of mice hindlimbs was assessed using a rip strength meter equipped with a pull bar. To measure grip strength, mice' paws were placed on a pull bar and gently pulled until the single mouse hindlimb released the bar (Bioseb, Virtolles CEDEX, France) after pre-determined periods of post-transplantation of P, PC, PM, and PCM. Subsequently, the maximum strength of the grip prior to grip release is recorded.

S1.15 Statistical analysis

All variables were tested in three independent cultures for each experiment, which was repeated twice ($n = 6$). All experimental results are presented as mean ± standard deviation (SD). The data were tested for homogeneity of variance using Levene's test prior to statistical analysis. Statistical comparisons were performed using one-way analysis of variance (ANOVA) or Student's t-test. The statistical significance was as follows: * $p < 0.05$, ** $p < 0.01$, *** $p < 0.001$, **** $p < 0.0001$.

S2 Supplementary Table and Figures

Table S1 Sequences of primers used for qRT-PCR

Primer	Forward sequence	Reverse sequence
Myostatin	agtggatctaaatgagggcagt	gtttccaggcgcagcttac
MyoD	ggctacgacaccgcctactac	aaatcgcattggggtttgag
MyoG	ctacaggccttgctcagctc	acgatggacgtaagggagtg
MHC1	caatcaggaaccttcggaacac	gtcctggcctctgagagcat
PGC-1α	agccgtgaccactgacaacgag	gctgcatggttctgagtgctaag
MHC4	caagtcatcggtgtttgtgg	tgtcgtacttgggagggttc
iNOS	caccttggagttcacccagt	accactcgtacttgggatgc
SGK1	tggcacagtagaatccacca	tccacttccaccgtcttacc
AKT	caccttggagttcacccagt	accactcgtacttgggatgc
PI3K	tggcacagtagaatccacca	tccacttccaccgtcttacc
mTOR	aactttggcattgtggaagg	acacattgggggtaggaaca
GAPDH	aactttggcattgtggaagg	acacattgggggtaggaaca

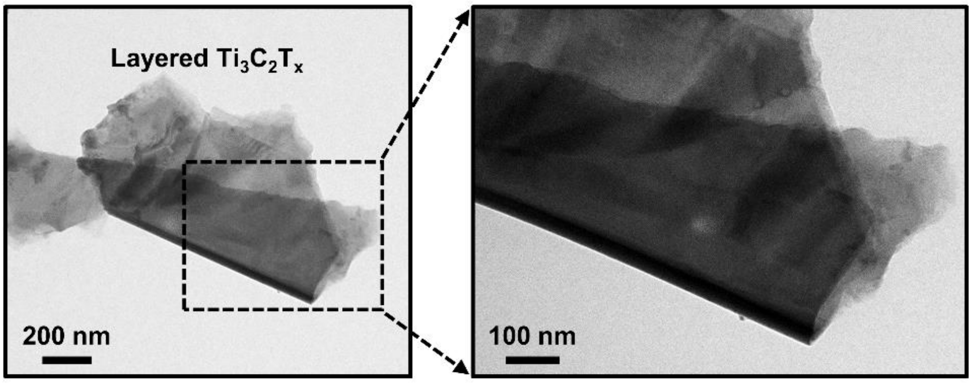

Fig. S1 Highly magnified TEM images of few-layered MXene NPs

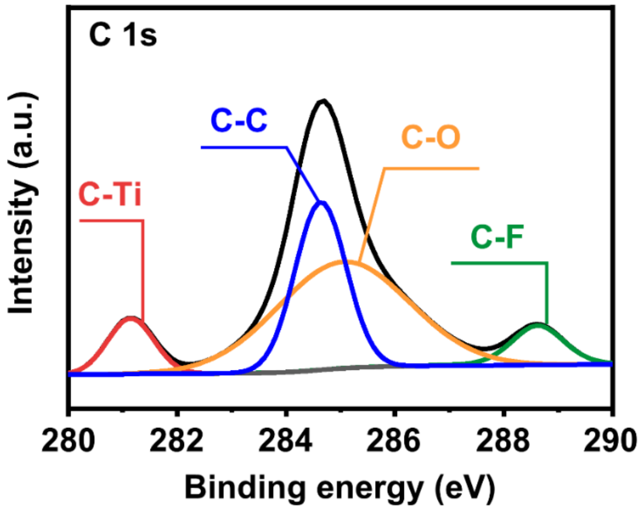

Fig. S2 High-resolution XPS spectrum for C 1s in $Ti_3C_2T_x$ MXene NPs

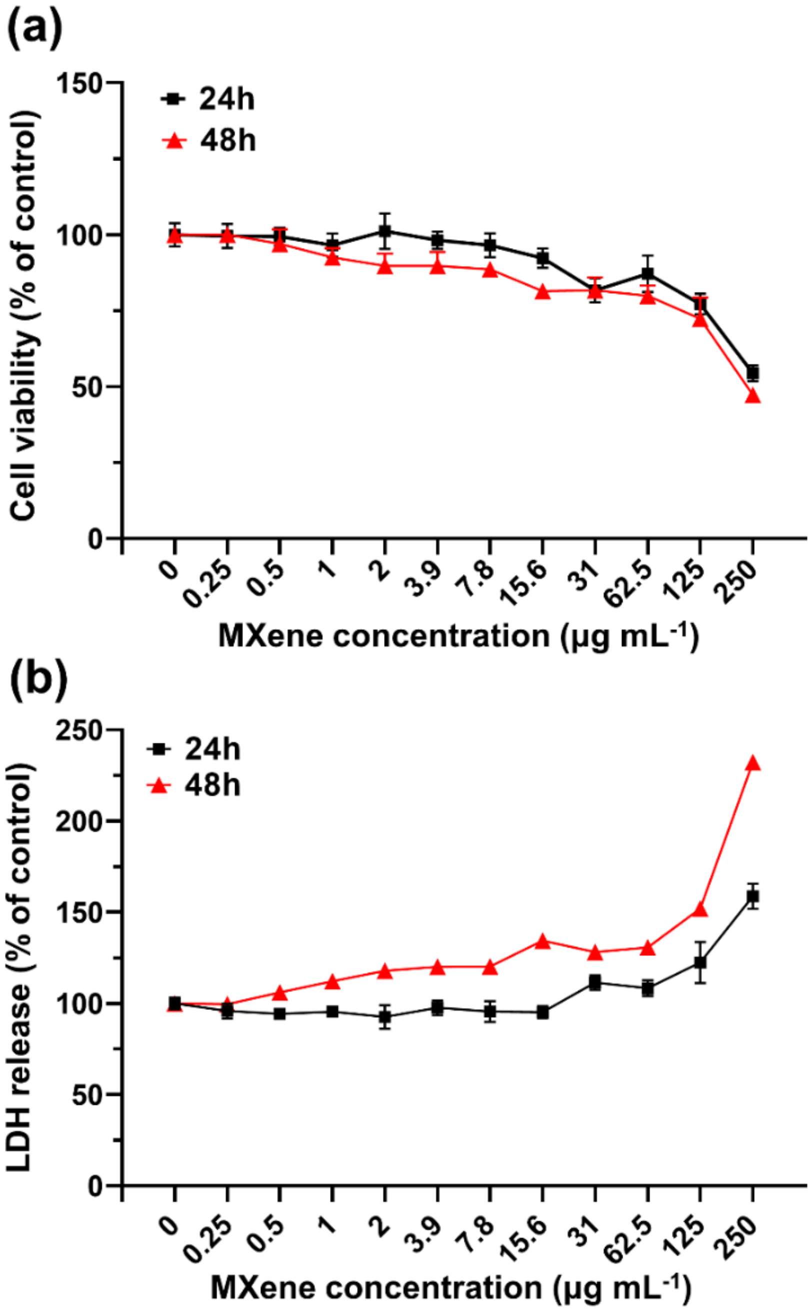

Fig. S3 Dose-dependent cytotoxicity of MXene NPs on C2C12 myoblasts. **a** CCK-8 assay results showing cell viability. **b** LDH release assay results indicating membrane integrity. The data are expressed as the mean ± SD (n = 6)

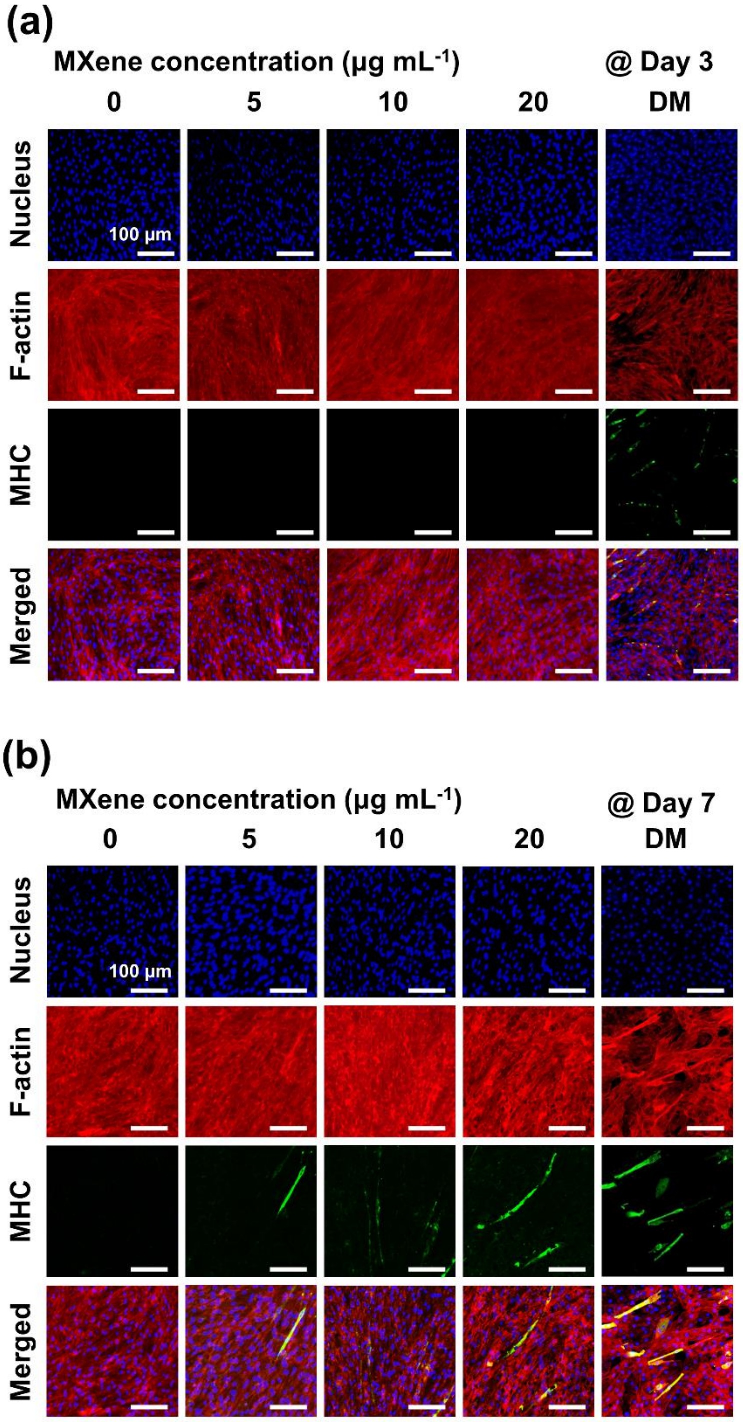

Fig. S4 Immunocytochemical analysis on C2C12 myoblasts. **a** Three days and **b** seven days of incubation with 0, 5, 10, and 20 μg mL^{-1} of MXene NPs treatment or DM condition. The F-actin and nucleus were stained with TRITC-labelled phalloidin (red) and DAPI (blue), while the MHC was stained with FITC (green). All fluorescence images were obtained from representative portions of the samples. Scale bars denote 100 μm

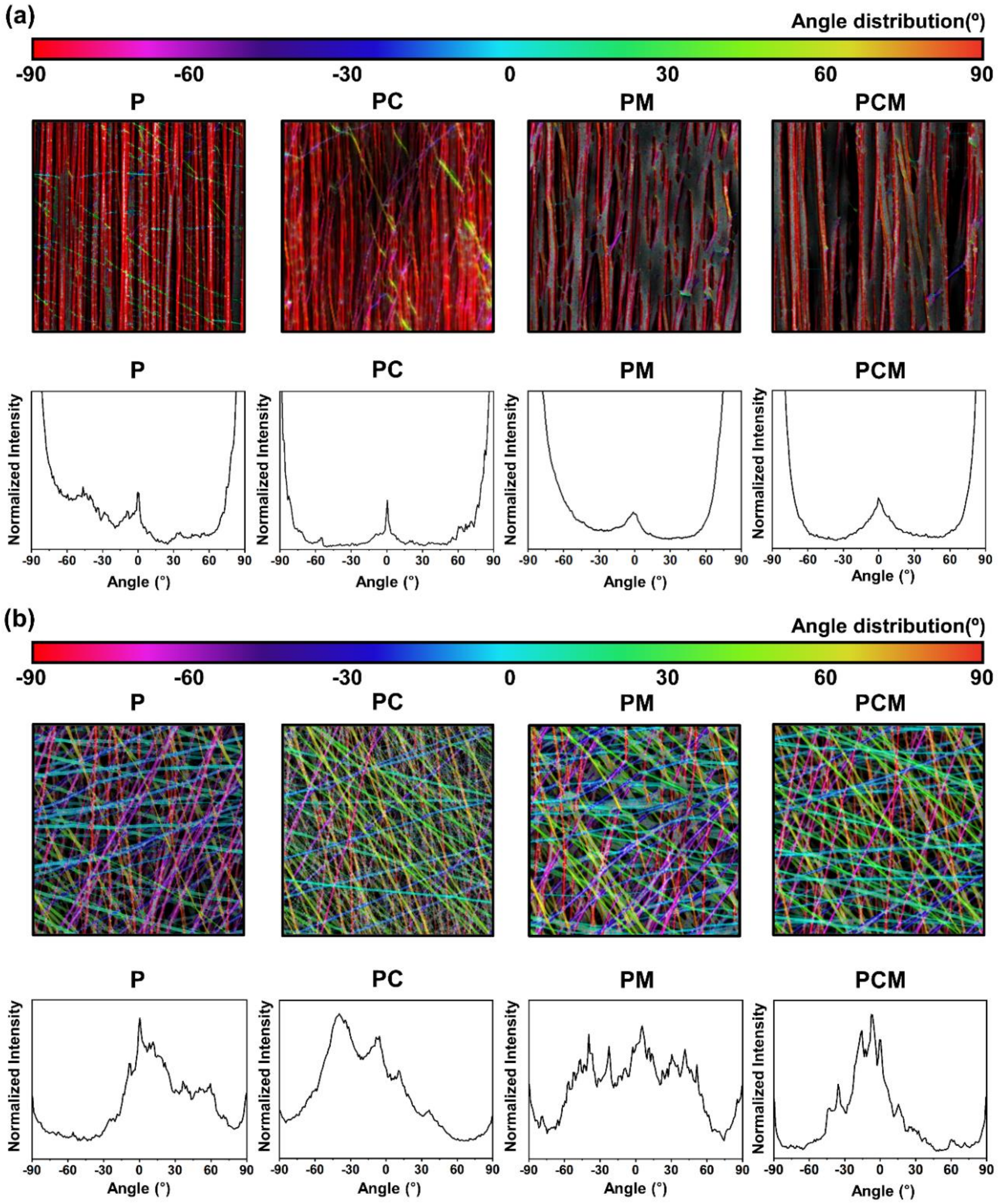

Fig. S5 Quantification of fiber orientation. **a** Aligned nanofibrous matrices and **b** randomly oriented nanofibrous matrices. The FE-SEM images of different matrices were processed by FFT mode using ImageJ. The normalized intensities were calculated based on the color maps

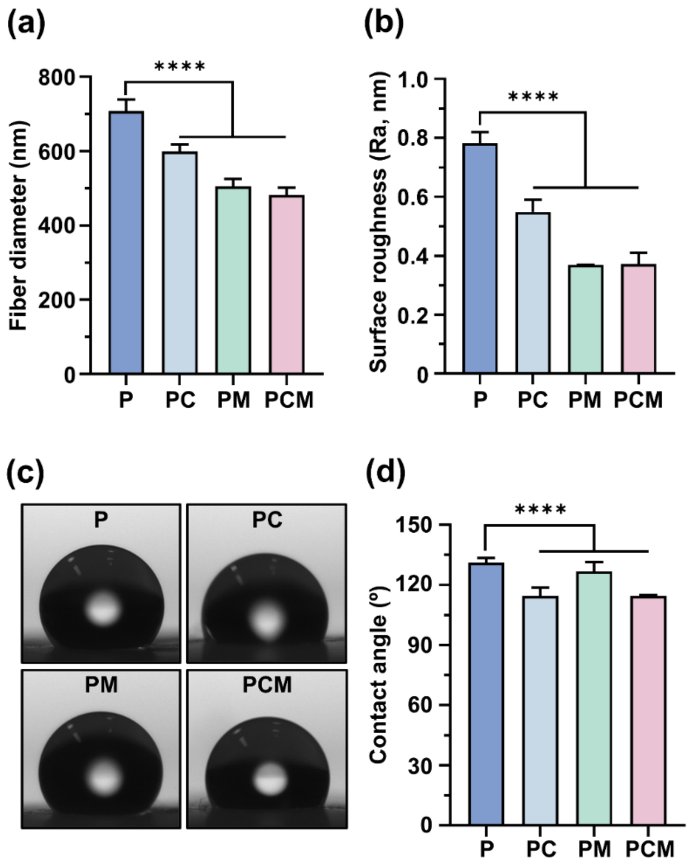

Fig. S6 Physicochemical characterization of randomly oriented nanofibrous matrices. **a** Average diameter of nanofibers based on FE-SEM images, **b** Surface roughness characterized by AFM images, **c** contact angle measurements, and **d** their quantified data. The data are expressed as the mean ± SD (n = 6). Asterisks (****) denote significant differences compared to the control (**** $p < 0.0001$)

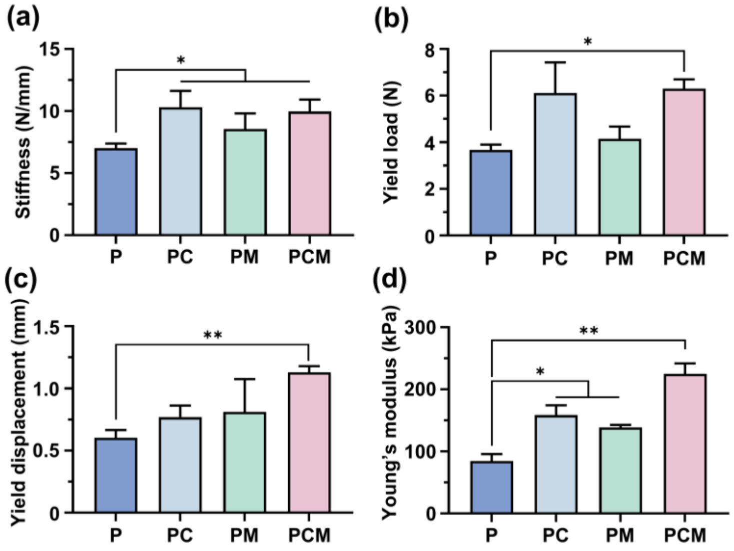

Fig. S7 Mechanical properties of nanofibrous matrices indicating **a** stiffness, **b** yield load, **c** yield displacement, and **d** Young's modulus. The data are expressed as the mean ± SD (n = 6). Asterisks (* and ***) denote significant differences compared to the control (* $p < 0.05$ and ** $p < 0.01$)

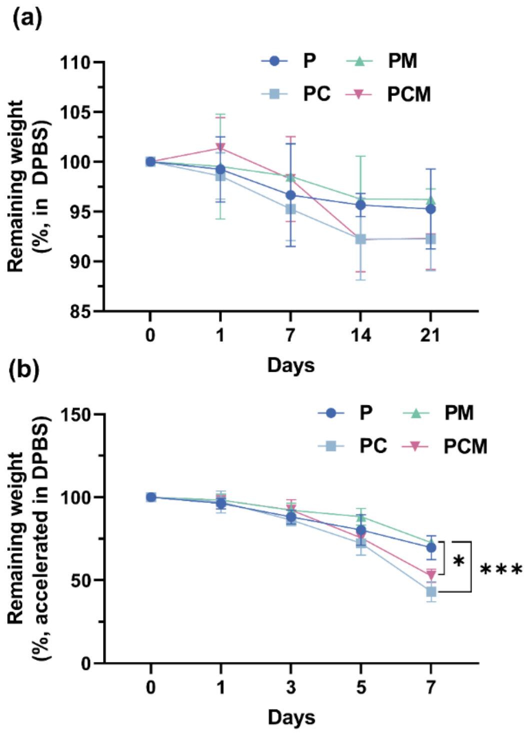

Fig. S8 Degradation property of nanofibrous matrices. Remaining weight in **a** body temperature (37°C) and **b** accelerated condition (70°C). The data are expressed as the mean ± SD (n = 6). Asterisks (* and ***) denote significant differences compared to the control (* $p < 0.05$ and *** $p < 0.001$)

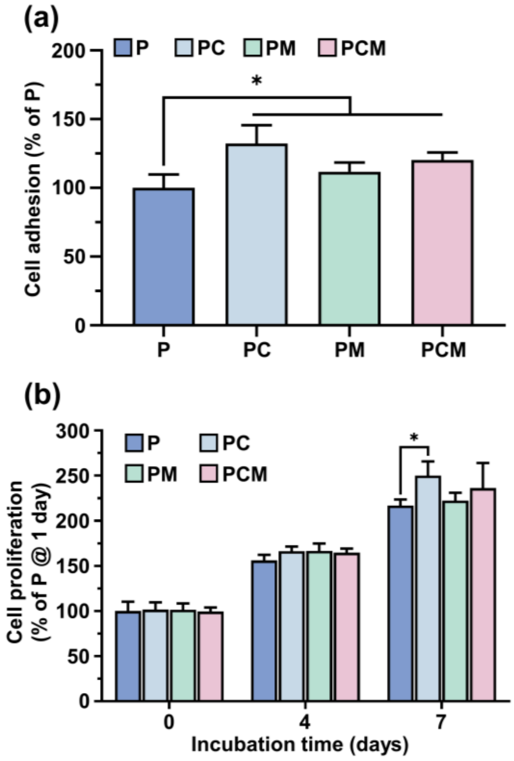

Fig. S9 Cell behaviors on randomly oriented nanofibrous matrices. **a** Adhesion and **b** proliferation of C2C12 myoblasts. The data are expressed as the mean ± SD (n = 6). Asterisk (*) denotes significant difference compared to the control (* $p < 0.05$)

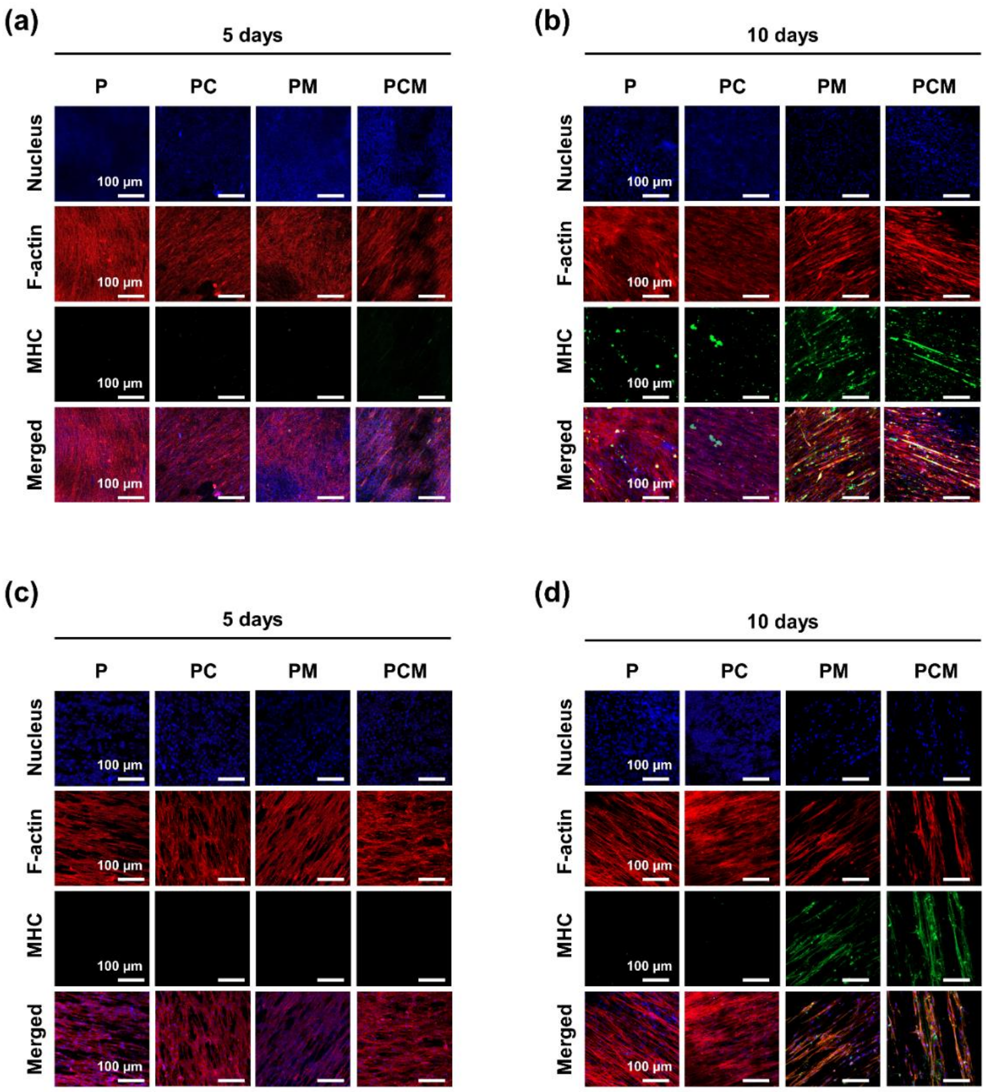

Fig. S10 Immunocytochemical analysis on C2C12 myoblasts cultured on randomly oriented or aligned nanofibrous matrices. **a** Five and **b** ten days cultured cells on randomly oriented nanofibrous matrices. **c** Five and **d** ten days cultured cells on aligned nanofibrous matrices. Increased cell arrangement and MHC expression were observed in the aligned nanofibrous matrices. The F-actin and nucleus were stained with TRITC-labelled phalloidin (red) and DAPI (blue), while the MHC was stained with FITC (green). All fluorescence images were obtained from representative portions of the samples. Scale bars denote 100 μm

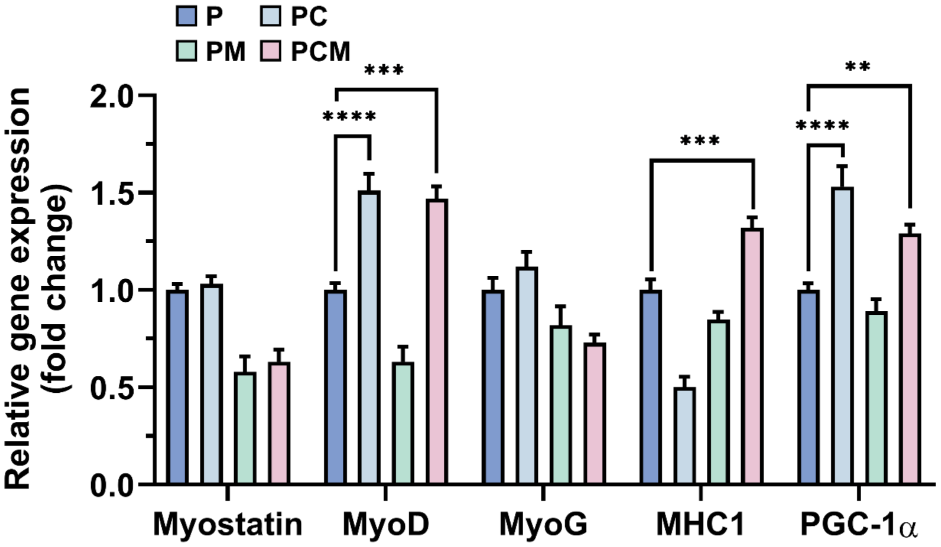

Fig. S11 qRT-PCR results of C2C12 myoblasts cultured on randomly oriented nanofibrous matrices. The data are expressed as the mean ± SD (n = 6). Asterisks (**~****) denote significant differences compared to the control (** $p < 0.01$, *** $p < 0.001$, and **** $p < 0.0001$)

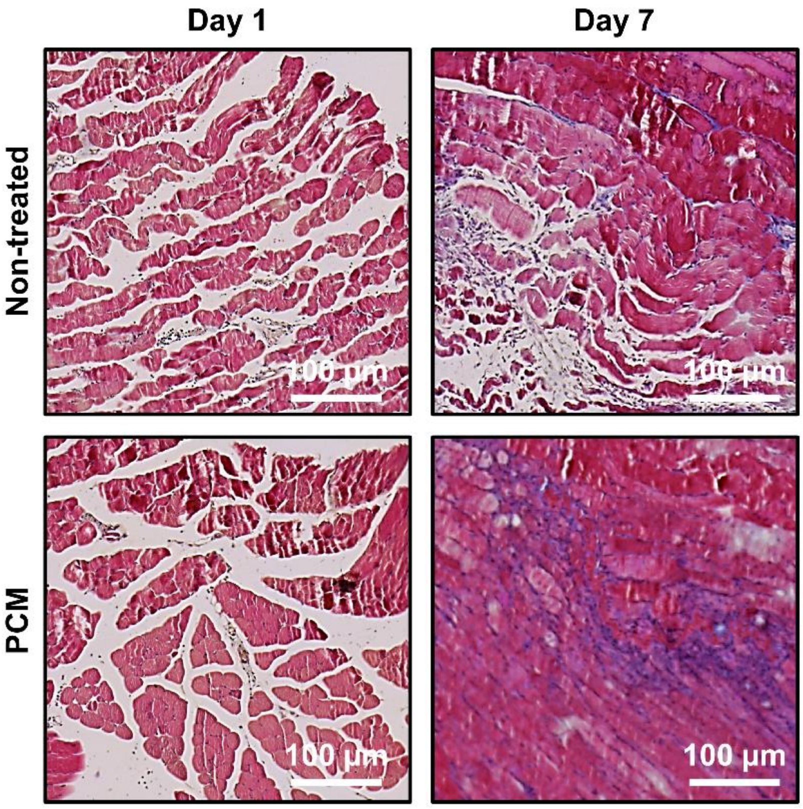

Fig. S12 Skeletal muscle tissue was obtained at 1 and 7 days after VML with Masson's trichrome staining. All images were obtained from representative portions of the samples. Scale bars denote 100 μm

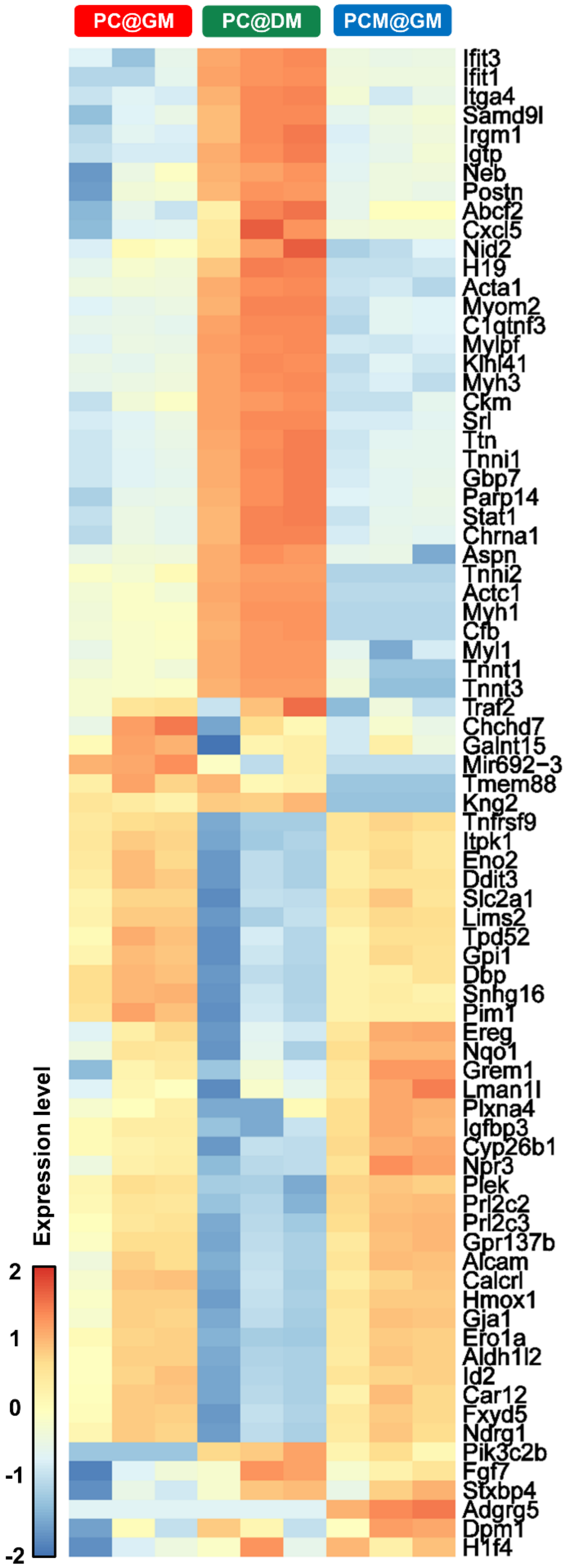

Fig. S13 Detailed names of genes of Fig. 6e

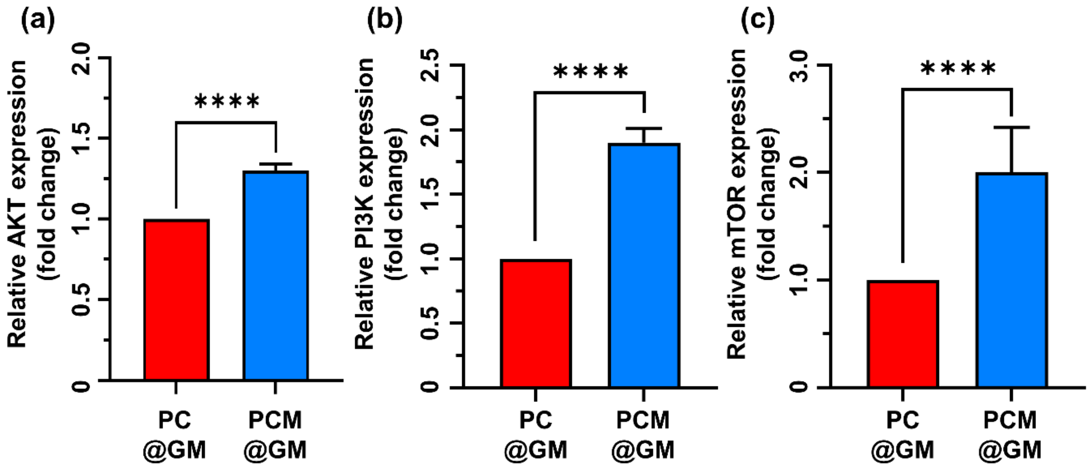

Fig. S14 AKT, PI3K and mTOR expression levels during the myogenic differentiation of C2C12 myoblasts were determined using qRT-PCR in PCM@GM compared with the PC@GM group. The data are expressed as the mean ± SD (n = 6). Asterisks (****) denote significant differences compared to the control (**** $p < 0.0001$)

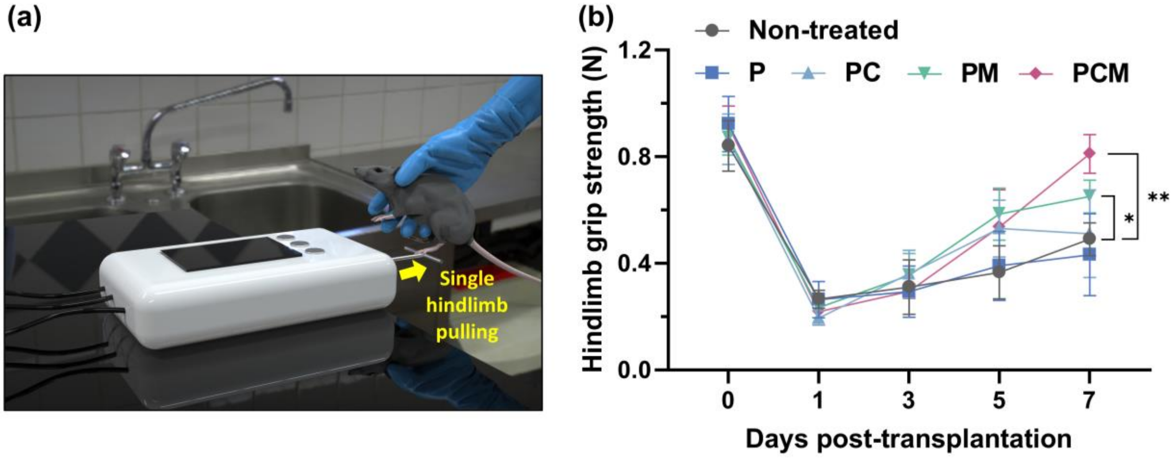

Fig. S15 Grip test showing single hindlimb grip strength 1 to 7 days after transplantation of nanofibrous matrices. (a) Schematic illustration of the grip test and (b) grip strength of the mice. The data are expressed as the mean ± SD (n = 6). Asterisk (*) denote significant differences compared to the value before surgery (at 0 day, * $p < 0.05$ and ** $p < 0.01$)
